# The central role of IL-6 in the differential effects of GLP-1 receptor agonists and metformin across multiple health conditions

**DOI:** 10.3389/fimmu.2026.1864104

**Published:** 2026-07-21

**Authors:** Zeev Elkoshi

**Affiliations:** Retired, Kfar Saba, Israel

**Keywords:** cancer, cardiovascular disease, GLP-1 receptor agonists, metabolic dysfunction–associated steatohepatitis, metabolic dysregulation, metformin, osteoarthritis, polycystic ovary syndrome

## Abstract

An earlier publication suggested that elevated TGF-β1 together with IL-1β and IL-6 drives neutrophilic asthma, increases cancer risk, and is associated with metabolic dysregulation. This article examines how these cytokines shape the therapeutic efficacy and safety profiles of GLP-1 RAs compared with metformin. Integrating published data, stronger suppression of *circulating* IL-6 likely contributes to the superior efficacy of GLP-1 RAs in lowering blood glucose, improving insulin sensitivity in patients with polycystic ovary syndrome (PCOS), reducing cardiovascular risk, and reducing osteoarthritis-related perioperative infections, but not to their superior weight-loss effects. Superior weight loss, which is IL-6 independent, further enhances the efficacy of GLP-1 RAs in improving metabolic dysfunction–associated steatohepatitis (MASH), PCOS, and survival in cancer patients. Systemic IL-6 suppression may also explain the higher frequency of allergic adverse effects with GLP-1 RAs, whereas local interference with IL-6 signaling in the gastrointestinal tract, shared by both agents, likely accounts for frequent gastrointestinal adverse events reported with both. Although metformin may be more effective in improving asthma outcomes in the general asthma population, GLP-1 RAs are likely to be more effective in neutrophilic asthma. Metformin also reduces osteoarthritis-related joint pain, consistent with the limited efficacy of IL-6R inhibition in reducing this pain. Finally, the lack of effect of IL-6 pathway inhibitors on cancer incidence may explain the similar efficacy of GLP-1 RAs and metformin in reducing the risk of most cancers.

## Graphical Abstract

**Figure:**
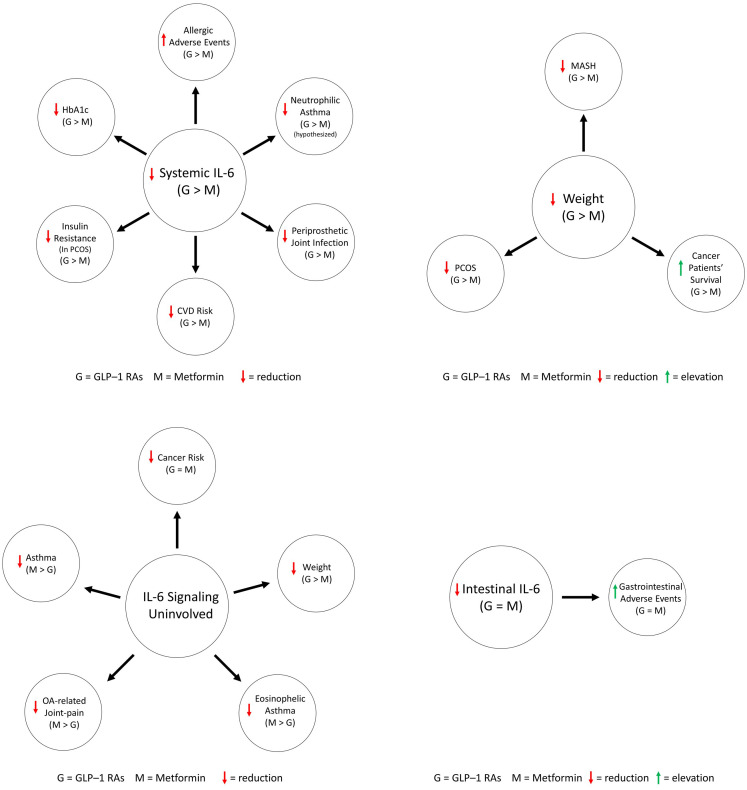

## Introduction

Glucagon-like peptide-1 receptor agonists (GLP-1RAs), initially introduced for type 2 diabetes (T2D), have increasingly become central to medical obesity treatment. In clinical trials, these agents frequently achieve 15–20% reductions in body weight, marking a substantial therapeutic impact. The regulatory authorization of liraglutide, semaglutide, and tirzepatide for long-term weight management has further broadened their clinical application (1). Multiple large cardiovascular outcomes trials have shown that GLP-1 RAs lower the incidence of major adverse cardiovascular events (MACE) in patients with T2D who are at elevated cardiovascular risk (2). In March 2024, FDA approved Wegovy^®^ (semaglutide injection) in combination with a reduced calorie diet and increased physical activity to reduce the risk of major adverse cardiovascular (CV) events in adults with established CV disease and either obesity or overweight (3, 4). In the recent ESSENCE trial, 62.9% of patients receiving semaglutide achieved resolution of metabolic dysfunction-associated steatohepatitis without worsening of fibrosis at 72 weeks, compared to 34.3% in the placebo group (5). In August 2025, the U.S. Food and Drug Administration approved injectable semaglutide 2.4 mg (Wegovy^®^) for the treatment of noncirrhotic metabolic dysfunction–associated steatohepatitis (MASH; formerly known as nonalcoholic steatohepatitis) in adults with moderate-to-advanced liver fibrosis (6). Beyond their effects on glycemic control, weight loss, cardiovascular disease, and MASH, GLP-1 RAs are now being evaluated for a range of other conditions, including chronic kidney disease (CKD), obstructive sleep apnea, knee osteoarthritis, polycystic ovary syndrome (PCOS), neurodegenerative disease, substance abuse disorders (1), and asthma (7–9).

An earlier publication proposed a binary model describing the concentration-dependent actions of TGF-β1 (high versus low), particularly in the presence of IL-6 and IL-1β, demonstrating that elevated TGF-β1 promotes neutrophilic asthma and metabolic dysregulation, increases cancer risk, and suppresses allergic rhinitis (10). The present work examines the roles of these three cytokines in shaping the efficacy and safety profiles of GLP-1 RAs compared with metformin.

## GLP-1 RAs downregulate IL-1β, IL-6, and the TGF-β/Smad signaling pathway in T2D patients

A recent meta-analysis of 52 randomized clinical trials (RCTs) reported significantly lower circulating IL-1β and IL-6 levels in patients with T2D treated with GLP-1 RAs, compared with placebo or conventional diabetes therapies including lifestyle intervention, sitagliptin, insulin, metformin, acarbose, glimepiride, pioglitazone, glibenclamide, and combinations of these interventions (11).

Numerous preclinical studies demonstrate that GLP-1 RAs suppress TGF-β/Smad signaling. For example, semaglutide downregulated hepatic TGF-β/Smad pathway activity in a murine model (12), while liraglutide inhibited myocardial fibrosis in rats by suppressing Smad signaling and reducing cardiac fibroblast activation and extracellular matrix secretion (13). Peng et al. (14) demonstrated in a rat kidney transplant model that dual GLP-1 and glucagon receptor agonism ameliorates allograft fibrosis by downregulating fibrosis-associated markers, including TGF-β1. In a metabolic disease model, dulaglutide was shown to reduce the expression of inflammatory cytokines (IL-1β, TNF-α, IL-6) as well as TGF-β1 mRNA levels (15). Suppression of TGF-β/Smad signaling by GLP-1 RAs has also been demonstrated in *in vitro* systems. In human normal colon epithelial cells (NCM-460) and colorectal cancer cell lines (LoVo and HCT116), liraglutide modulated the TGF-β/Smad3 pathway by reducing TGF-β expression, decreasing the phosphorylated-Smad3/Smad3 ratio, and lowering N-cadherin levels, while increasing E-cadherin expression (p < 0.05) (16). In addition, liraglutide was evaluated in a unilateral ureteral obstruction (UUO) model of renal fibrosis, as well as in recombinant TGF-β1–induced EMT in rat renal tubular epithelial cells (NRK-52E). Liraglutide treatment reduced the expression of TGF-β1 and its receptor (TGF-β1R) and inhibited activation of downstream signaling molecules, including phosphorylated Smad3 and phosphorylated ERK1/2 (17).

## Metformin selectively downregulates IL-1β and TGF-β/Smad signaling in T2D patients, but not circulating IL-6

Metformin inhibits mitochondrial NADH:ubiquinone oxidoreductase (complex I). It has been demonstrated that inhibition of this complex in macrophages reduces lipopolysaccharide (LPS)-induced IL-1β production while enhancing IL-10 expression (18). Subsequently, metformin was shown to attenuate *il1b* transcript levels during the acute LPS response through activation of AMPK, independently of IL-10, HIF-1α, and NF-κB signaling (19). In addition, metformin inhibited IL-1β–induced release of the pro-inflammatory cytokines IL-6 and IL-8 in endothelial cells, smooth muscle cells, and macrophages, in a dose-dependent manner (20). A small study including 117 patients with T2D compared individuals treated with metformin with those not receiving metformin and found that IL-1β mRNA levels were significantly lower in the metformin group (21). However, the study does not clarify whether metformin was used as monotherapy, which concomitant medications were taken by either group, or whether the control group included drug-naïve patients. Therefore, it is not possible to attribute the observed difference in IL-1β expression specifically to metformin.

Metformin suppresses IL-6 expression under inflammatory conditions in both macrophages (22, 23) and endothelial cells (20). In addition, metformin has been reported to attenuate IL-6 signaling by downregulating IL-6 receptor expression on multiple myeloma cells (24). However, a meta-analysis of 13 randomized controlled trials including 1776 participants found that metformin had no significant effect on the circulating levels of IL-6 in patients having impaired glucose tolerance or T2D (25).

Metformin have shown to antagonizes TGF-β1 signaling via a direct interaction with TGF-β1, to inhibits the binding probability of TGF-β1 and TβRII, and to inhibit TGF-β1-induced TβRII dimerization in mouse embryonic fibroblast and human colorectal carcinoma epithelial cell lines (26). It was also demonstrated in rat model and *in vitro*, that metformin prevents peritendinous fibrosis by inhibiting TGF-β signaling, and rats treated with metformin showed significantly decreased phosphorylation levels of smad2/3 (27).

These results are summarized in **Table 1**.

**Table 1:** The effects of GLP-1 RAs and metformin on IL-1β, IL-6, and TGF-β1.

Drug	IL-1β	IL-6	TGF-β1
GLP-1 RAs	Significantly reduce circulating IL-1β (meta-analysis of 52 RCTs) (11)	Significantly reduce circulating IL-6 (meta-analysis of 52 RCTs) (11)	Suppress TGF-β/Smad signaling in rodent models (12 –17)
Metformin	Reduces IL-1β production in macrophages and attenuates *il1b* transcription during LPS stimulation *in vitro* (19). Lower IL-1β mRNA levels in metformin-treated vs untreated T2D patients (methodology unclear) (21)	No significant effect on circulating IL-6 in patients with IGT or T2D (meta-analysis of 13 RCTs) (25)	Antagonizes TGF-β1 signaling *in vitro* (26) and *in vivo* (27)

IGT, impaired glucose tolerance; LPS, Lipopolysaccharides; mRNA, messenger ribonucleic acid; T2D, Type 2 diabetes; RCTs, randomized controlled trials.

## The role of IL-1β, IL-6 and TGF-β1 in metabolic dysregulation

Metabolic dysregulation refers to disturbances in the body’s normal metabolic functions, including energy generation, nutrient handling, and waste processing. When these processes are impaired, the result can be a range of clinical conditions, such as obesity, insulin resistance, glucose intolerance, T2D, dyslipidemia, and hypertension (28).

IL-1β: Interleukin 1β is a major pro-inflammatory factor involved in metabolic regulation. IL-1β is part of the obesity-associated chronic inflammation that can be detrimental to metabolic health. Inflammasome is a multimeric protein complex, which is activated by cell nutrients, such as glucose and free fatty acids, inducing IL-1β production. Pancreatic islet macrophage-derived IL-1β impairs insulin secretion, contributing to the development of glucose intolerance and T2D, and induce insulin resistance (29). However, in short, transient settings such as the postprandial state, IL-1β exerts the opposite effect by enhancing insulin secretion from β-cells (30). IL-1β is a potent inducer of IL-6 in fibroblasts, endothelial cells, keratinocytes, and peripheral blood monocytes (31). In non-diabetic obese individuals, anakinra, an IL-1 receptor (IL-1R) antagonist, significantly reduced C-reactive protein (CRP) without improving insulin sensitivity (32). In contrast, anakinra enhanced early insulin secretion in subjects with impaired glucose tolerance (33) and improved glycemia, β-cell function, and CRP in T2D patients (34), but the effects were small. Canakinumab, an anti-IL-1 monoclonal antibody (mAb), improved early insulin secretion rate (relative to glucose) versus placebo in insulin-treated patients, but results were statistically non-significant (35).

IL-6: Interleukin-6 is a key immunomodulatory cytokine that affects the pathogenesis of diverse diseases, including autoimmune diseases, chronic inflammatory conditions and cancer. IL-6 can induce its effect mainly through two distinct signaling pathways: the classical (cis) and the trans signaling (although a third pathway termed cluster signaling was also reported). Classical signaling involves two membrane-bound components: the IL-6 receptor, which is expressed on a limited number of cell types including hepatocytes and certain leukocytes, and the glycoprotein gp130, which is widely expressed. The classical pathway predominantly triggers an anti-inflammatory response (36). This anti-inflammatory reaction involves an activation of the transcription factor STAT3 in immune and non-immune cells. It results in increased intestinal cell proliferation, reduced apoptosis, suppression of bacterial infections, and induction of the hepatic acute-phase response. The trans-signaling IL-6 pathway, on the other hand, involves soluble IL-6 receptor (sIL-6R), and the ubiquitous gp-130. It exerts its effect on many body cells (since bound IL-6 receptor is not required). Trans-signaling mediates a pro-inflammatory response which include recruitment of mononuclear cells, inhibition of T cell apoptosis, and inhibition of Tregs differentiation (37). Under homeostasis, the trans-signaling effect of IL-6 is buffered by a complex of sIL-6R and soluble glycoprotein 130 (sIL-6R-sgp130) which competes with the complex IL-6-sIL-6R-gp130, and actually neutralizes trans-signaling due to stronger affinity. Under chronic and acute inflammation, sIL-6R concentration triples while sgp130 concentration does not increase which results in an overload of IL-6R-sgp130 buffer capacity. This allows systemic trans-signaling and a pro-inflammatory response (38). Muscle contraction, similar to inflammation, releases IL-6 into the circulation. A single bout of acute exercise resulted in a 5-fold increase (P<0.05) in IL-6. However, the increase in sIL-6R was only 1.2 (P<0.05) (39). During fatiguing submaximal exercise in humans, circulating IL-6 increased by 75.7% (P< 0.05) during the exercise period while both sIL-6R and sgp130 increased only by 9.6% (P<0.05) (40). These results demonstrate that although IL-6 concentration increases following exercise, the increase in sIL-6R is modest and sgp130 is not affected by exercise. Therefore, buffer effect of sgp130 is preserved following exercise and IL-6 released by muscle contraction mediates its effect through the classical signaling pathway with the result of an anti-inflammatory effect. In a review of 24 studies investigating the effects of exercise training on chronic inflammation in obesity, post-exercise CRP is either reduced or remain unchanged. This review concludes that current evidence indicates that structured exercise training—including aerobic and resistance modalities—attenuates chronic inflammation, particularly in individuals with obesity who exhibit elevated inflammatory biomarkers and participate in longer-term interventions. However, in addition to myokine release following muscle contraction during exercise, several other mechanisms contribute to the anti-inflammatory effect of exercise in obese individuals (33, 41).

The metabolic syndrome is associated with low-grade inflammation and accumulation of white adipose tissue. During this chronic inflammation, IL-6 produced by M1 type adipose tissue macrophages induce a pro-inflammatory reaction. Additionally, muscle contraction associated with exercise stimulates the release of myokines, including IL-6, that can have favorable effects on lipid metabolism and inflammatory pathways. IL-6 released during physical activity stimulates GLP-1 secretion by intestinal L-cells and promotes the reprogramming of pancreatic α-cells to produce GLP-1 through upregulation of prohormone convertase 1/3. Consequently, exercise-induced IL-6–driven GLP-1 supports metabolic adaptation by reducing gut motility and suppressing appetite. However, IL-6 shows inconsistent effects on insulin signaling: while several studies indicate that IL-6 promotes insulin resistance, others suggest that it exerts insulin-like effects and enhances insulin sensitivity (29). Diabetic retinopathy is a complication of diabetes that may develop to a sight-threatening stage. IL-6 is a key cytokine associated with chronic retinal inflammation which characterizes this condition (42). A key mediator of diabetic retinopathy is vascular endothelial growth factor (VEGF), which may be directly induced by IL-6 (as well as IL-1β) (43). Polycystic ovary syndrome and insulin resistance are two metabolic conditions associated with the metabolic syndrome. (a) Polycystic ovary syndrome is a common disorder characterized by androgen excess and ovarian dysfunction, typically presenting with hirsutism, anovulation, and infertility. PCOS is strongly associated with an increased risk of diabetes. Weight management is a first-line treatment for women with PCOS during and independent of pregnancy (44). In a meta-analysis evaluating inflammatory markers in women with PCOS, circulating IL-6 levels were reported in 16 studies: five showed significantly elevated IL-6 in women with PCOS compared with healthy controls, whereas the remaining eleven found no significant difference (45). In a review focusing on the role of IL-6 signaling in PCOS, the majority of the 17 clinical studies reviewed reported elevated IL-6 levels in PCOS patients with an obese phenotype (46). However, a meta-analysis of 10 studies revealed no statistically significant differences in IL-6 levels between PCOS and controls (47).

In a preclinical study, IL-6 was markedly correlated with total cholesterol, low density lipoproteins, triacylglycerol, visceral fat mass and body weight (48). In a recent clinical study, elevated serum levels of interleukin 6 in non-smoking perimenopausal and postmenopausal women were associated with hypertriglyceridemia and insulin resistance (49). Elevated IL-6 levels were also observed in the aqueous humor of diabetic patients compared with non-diabetic patients, and these levels correlated with the severity of diabetic retinopathy (42). In all these studies IL-6 was predefined as an end-point. It must be realized however that association between events does not imply causality. Indeed, in a systematic review of 9 studies including 1531 patients (mostly patients with rheumatoid arthritis), the use of IL-6 pathway inhibitors was associated with weight gain (50). It was also reported that IL-6 signaling may protect from excess fat accumulation by stimulating thermogenesis in adipocytes (51).

There is no clear evidence indicating the involvement of IL-6 signaling in PCOS (46 Borthakur); (b) Insulin resistance (IR) is a state in which normal insulin levels fail to produce an adequate biological response in target tissues. One hour after infusion, tocilizumab, an anti-IL6 inhibitor, decreased insulin resistance and increased insulin sensitivity in non-diabetic patients with rheumatoid arthritis (RA) refractory to methotrexate (52). A small clinical study (11 non-diabetic patients with rheumatoid diseases) found that tocilizumab treatment over 3 months improved insulin sensitivity measures after therapy, compared with baseline (53). Treatment with tocilizumab significantly lower HbA1c values in patients with rheumatoid arthritis compared to baseline levels (54). In another study, the use of anti-IL6 drugs (tocilizumab and sarilumab) has been shown to lead to a further improvement in HbA1c values compared to the use of other classes of biologic disease-modifying anti-rheumatic drugs (bDMARD) (55).

TGF-β1: Yadav et al. (56) highlighted a key role for the TGF-β/Smad3 signaling pathway in the control of glucose and energy balance. Their work showed that mice lacking Smad3 are resistant to diet-induced obesity and diabetes. Moreover, the study reported a strong association between TGF-β1 levels and adiposity in both rodents and humans. It further showed that TGF-β1, together with other members of the TGF-β superfamily, interferes with normal adipocyte development.

Pancreatic β-cell dysfunction or loss is a fundamental pathological hallmark shared across all forms of diabetes mellitus. The TGF-β/Smad signaling pathway has emerged as a critical regulator of β-cell physiology. Notably, Smad3 deletion has been shown to simultaneously enhance β-cell function, reduce β-cell apoptosis, and improve systemic insulin sensitivity, effectively preventing overt diabetes in diabetic mouse models (57–59).

Accumulating evidence indicates that the TGF-β signaling pathway is centrally involved in the pathogenesis of PCOS (60).

It has shown that peripheral blood monocytes from hypertensive patients display increased TGF-β production and gene expression (61). Moreover, these individuals often present with elevated circulating levels of proinflammatory cytokines, including IL-1β and IL-6 (62).

## The role of IL-1β, IL-6, and TGF-β1 in MASH

IL-1β: A recent preclinical study demonstrated that, in mice fed a Western diet, IL-1β produced by macrophages drives hepatic inflammation and fibrosis by inducing chemokine expression in liver sinusoidal endothelial cells via IL-1R1 signaling (63). In another preclinical study, a MASH diet combined with alcohol was shown to promote hepatic recruitment of macrophages and neutrophils and to induce IL-1β production via NLRP3 activation in these immune cells. The resulting IL-1β acted on hepatocytes to stimulate CXCL1 and LCN2 expression, which in turn drove further neutrophil infiltration and activation in the liver (64).

IL-6: The evidence regarding the role of IL-6 in MASH is conflicting. Hepatocytes are among the major non-immune cell types that express membrane-bound IL-6 receptor, rendering them responsive to classical IL-6 signaling. In hepatocytes, classical IL-6 signaling activates STAT3 and induces the acute-phase response. Acute-phase proteins are plasma proteins whose concentrations either increase (positive acute-phase proteins) or decrease (negative acute-phase proteins) during inflammation. Positive acute-phase proteins serve diverse functions within the innate immune response, including inhibition of microbial growth, modulation and negative feedback of inflammatory processes, and regulation of coagulation. Collectively, these acute-phase proteins contribute to containment of tissue damage and limitation of the underlying insult, whether caused by infection, or organ injury (65, 66),.

Preclinical studies indicate a context-dependent role for IL-6 in metabolic liver disease. IL-6 deficiency or hepatocyte-specific IL-6Rα deletion has been linked to hepatic inflammation and systemic insulin resistance, supporting a protective role for hepatic IL-6 signaling in metabolic homeostasis (67, 68). IL-6 also enhances central leptin responsiveness and resistance to diet-induced obesity in mice (69), and genetic proxying of reduced IL-6 receptor signaling has been associated with increased MASH risk (70).

Conversely, systemic IL-6 neutralization improved insulin resistance in certain diabetic models (71), while IL-6 deficiency reduced lobular inflammation in diet-induced steatohepatitis despite similar steatosis (72). Inhibition of IL-6/GP130 signaling has been reported to worsen steatosis yet attenuate liver injury, suggesting divergent effects on lipid accumulation and inflammation (73, 74). IL-6 receptor antagonism has also ameliorated pancreatic injury in MASH models (75).

Collectively, these findings highlight the dual nature of IL-6 signaling. Classical hepatocyte IL-6R signaling may exert protective effects, whereas trans-signaling may promote inflammation. The net hepatic outcome likely depends on experimental context and the degree of pathway inhibition.

TGF-β1: TGF-β1 contributes to the pathogenesis of metabolic dysfunction–associated steatotic liver disease (MASLD) primarily through activation of hepatic stellate cells, thereby promoting hepatic fibrosis. TGF-β1 produced by hepatic cells enhances collagen synthesis and drives fibrogenesis within the liver (76). Circulating TGF-β1 levels are significantly elevated in patients with MASLD compared with healthy controls (77). Mechanistically, TGF-β has been shown to increase the stability of α1(I) collagen mRNA in hepatic stellate cells, thereby augmenting collagen production (78). Smad3 has been identified as a central mediator of the fibrogenic response in NAFLD (non-alcoholic fatty liver disease, now termed MASLD) (79). Beyond its role in fibrosis, TGF-β1 can exacerbate hepatic inflammation by inducing TLR2 transcription and downstream target gene expression in macrophages via Smad3 signaling, contributing to disease progression in NASH (now termed MASH) (80).

## IL-1β, IL-6, and TGF-β1 promote neutrophilic but not eosinophilic asthma

Asthma comprises multiple phenotypes that can be broadly classified into eosinophilic and neutrophilic disease but includes also mixed, and paucigranulocytic phenotypes (81). Eosinophilic asthma, which affects approximately half of patients, is driven by Th2 cytokines (IL-4, IL-5, and IL-13), is characterized by elevated levels of allergen-specific IgE and is also referred to as “Type 2-high”. In contrast, neutrophilic asthma, which accounts for approximately 20%–30% of adult asthma cases (81, 82), is characterized by neutrophilic airway inflammation associated with IL-1β and IL-6 signaling, low Th2 activity, and reduced circulating IgE levels, and is often referred to as a “Type 2-low” asthma phenotype (83, 84). This phenotype involves IL-6 production by airway epithelial cells and neutrophils, IL-1β generated via inflammasome activation, and TGF-β derived from neutrophils and eosinophils. TGF-β, together with IL-1β and IL-6, IL-21, or IL-23, promotes Th17 differentiation (85), linking neutrophilic asthma to Th17-driven immunity. Moreover, preclinical studies showed that IL-6 signaling is essential for both the induction and maintenance of murine Th17 cells, as IL-6Rα–deficient Th17 cells rapidly lost their phenotype and failed to induce disease in two colitis models (86). Th17 cells produce IL-17, IL-21, and IL-22, with IL-17 driving neutrophil recruitment to the airways (87). Neutrophils further amplify inflammation by releasing TGF-β and promoting the processing of pro–IL-1β into active IL-1β (88).

## The role of IL-1β, IL-6, and TGF-β1 in osteoarthritis

IL-1β and IL-6 are among the most inflammatory mediators that drive osteoarthritis (OA) (89).

IL-1β: IL-1β exerts its main effect in OA – cartilage catabolic degradation- through mitogen-activated protein kinase (MAPK) signaling. IL-1β also stimulates the secretion of IL-6, which potentiates the catabolic effects of IL-1β and degrades cartilage by catabolism on its own. In addition, IL-1β induces the secretion of PGE-2 (prostaglandin E2), NO (nitric oxide) and COX-2 (cyclooxygenase-2), pro-inflammatory mediators that contribute to synovial inflammation, and enhance the secretion of IL-1β (a positive-feedback cycle). IL-1β also drives OA through NF-κB signaling, leading to the inhibition of type II collagen expression, increased production of matrix metalloproteinases and induction of IL-6 secretion (89).

IL-6: Although membrane bound IL-6R were detected on chondrocytes from OA patients (90), IL-6 trans-signaling is probably more important in OA (91). The role of IL-6 is hard to define in OA. On one hand it was shown that IL-6 and sIL-6R blocked IL-1-induced collagenolytic activity (92). In addition, upon aging, IL-6(-/-) male mice developed more severe spontaneous OA than controls (93). On the other hand, systemic inhibition of IL-6/Stat3 signaling has been shown to protect against experimental OA (94), and IL-6 acts as a mediator of HIF-2α-induced experimental OA cartilage destruction in mice (95). Moreover, IL-6, sIL-6R, or both reported to inhibit type II collagen production by rabbit articular chondrocytes through a transcriptional control (96). It was also reported that elevated IL-6 blood levels were associated with knee radiographic osteoarthritis and knee cartilage loss in elderly OA patients (97). In this study IL-6 was predefined as an endpoint.

TGF-β1: Chondrocytes engage two distinct TGF-β signaling pathways. The first, generally considered protective, signals through membrane-bound ALK5, leading to activation of cytoplasmic Smad2/3. These Smads associate with Smad4 and translocate to the nucleus, where they reprogram gene expression to promote anabolic and homeostatic responses. In contrast, a second catabolic pathway mediates TGF-β signaling via membrane-bound ALK1, resulting in activation of cytoplasmic Smad1/5/8. Following association with Smad4 and nuclear translocation, this pathway induces gene expression programs associated with cartilage degradation (98). This complexity of TGF-β signaling—where downstream effects and biological outcomes depend on the repertoire of available receptors—complicates prediction of the consequences of TGF-β targeting. In one study, intra-articular injection of TGF-β1 into the murine knee joint was reported to induce a strong and long-lasting stimulation of articular cartilage proteoglycan synthesis (99). However, the same group later reported that multiple intra-articular injections of TGF-β induced changes in articular cartilage and surrounding tissues resembling osteoarthritis in mice (100). The authors attributed the cartilage degeneration observed in the latter study to excessive and/or prolonged exposure to TGF-β, an interpretation that is further supported by *in vitro* findings (101).

## The role of IL-1β, IL-6, and TGF-β1 in cardiovascular pathology

IL-1β: IL-1 cytokines participate throughout the development of atherosclerosis. Both IL-1α and IL-1β are detectable within atherosclerotic plaques, where macrophages are the main producers. Their local release disrupts endothelial homeostasis by promoting oxidative stress, inducing pro-coagulant activity, and impairing vasodilation. Together, these effects contribute to plaque progression and enhance the risk of atherothrombosis (102).

IL-1β levels were found to be an independent risk factor predicting cardiovascular disease (CVD) risk in patients with newly diagnosed, drug naïve T2D (103). Canakinumab therapy (150 mg quarterly), which suppresses IL-1β–driven inflammation, produced a significant reduction in recurrent cardiovascular events compared with placebo, an effect unrelated to lipid lowering (104). A recent consensus by the American College of Cardiology recommends consideration of IL-1 blockade among select patients with multiple recurrent episodes of colchicine- and steroid-resistant pericarditis, with hsCRP levels >10 mg/L, in the absence of tuberculosis (105).

IL-6: IL-6 plays a pathogenic role in CVDs. It promotes the development of atherosclerosis, coronary artery disease, hypertension, arterial aneurysm, aortic dissection, cardiac fibrosis, myocarditis, and cardiac ischemia-reperfusion injury (106). In a double-blind, randomized, placebo-controlled, phase 2 trial, ziltivekimab, an anti-IL-6 antibody, markedly reduced biomarkers of inflammation and thrombosis relevant to atherosclerosis in a group of adults with elevated CRP and chronic kidney disease. At 12 weeks after randomization, median high-sensitivity CRP levels were reduced by 77% to 92%, depending on ziltivekimab dose, compared with 4% for the placebo group [107[. Another phase 2 study with ziltivekimab conducted in Japanese patients at high risk of atherosclerotic reported that “at end of treatment, median hsCRP levels were reduced by 96.2% in the 15 mg group (p < 0.0001 versus placebo), by 93.4% in the 30 mg group (p = 0.002 versus placebo), and by 27.0% in the placebo group” (108).

TGF-β1: Atherosclerosis, the pathological basis of coronary artery disease, is characterized by chronic vascular inflammation and dyslipidemia, whereas revascularization procedures such as stenting and bypass grafting cause acute vascular injury. Genetic deletion of TGFβ1, its receptors, or downstream signaling components underscores the essential role of TGFβ1 in vasculogenesis and vascular homeostasis. Accordingly, dysregulated TGFβ signaling is a hallmark of vascular disease and is associated with pathological vascular cell phenotypes, fibrosis, and smooth muscle cell remodelling. Many studies have identified an important role for the canonical Smad2/3 in driving intimal hyperplasia which can be viewed as an early, structural, and often initiating component of atherosclerosis (109, 110).

Pre-clinical models linked cardiac and vascular fibrosis to TGF-β signaling (111–114). Left ventricular dysfunction correlates with cardiac fibrosis (115, 116). Indeed, a recent study indicates a significant association between TGF-β1 levels and left ventricular diastolic dysfunction and arrhythmia risk in patients with early-onset coronary artery disease (117).

## The role of IL-1β, IL-6, and TGF-β1 in cancer

IL-1β (Cancer onset and early growth): In the context of chronic inflammation, IL-1β may directly promote the production of carcinogenic mediators, such as nitric oxide and reactive oxygen species. It has been shown that stomach-specific expression of human IL-1β in transgenic mice led to spontaneous gastric inflammation and cancer that correlated with early recruitment of myeloid-derived suppressor cells (MDSCs) to the stomach (118). Acting through IL-1R on epithelial cells, IL-1 directly promoted the malignant transformation of epithelial cells mediated by nuclear accumulation of NF-κB. In addition, IL-1 signaling in T cells triggered the secretion of pro-tumorigenic IL-17 and IL-22. Epithelial IL-1R1 deletion reduced tumorigenesis in the Adenomatous Polyposis Coli CRC model, supporting a cell-autonomous role of IL-1 signaling in early tumor outgrowth (119). In patients with atherosclerosis, treatment with canakinumab, an anti–IL-1β inhibitor, significantly reduced both lung cancer incidence and mortality (120) (note: the reduction in mortality may partly reflect the reduction in incidence).

IL-1β (Cancer propagation): (a) IL-1β pro-cancer role - IL-1β has been shown to drive tumor growth and metastasis *in vivo* and *in vitro* across multiple cancer types. A key mechanism involves suppression of antitumor immunity through recruitment of MDSCs, thereby promoting cancer cell proliferation and metastasis (121). IL-1β is essential for the differentiation of pathogenic Th17 cells in the gastrointestinal track (122). Th17 cells are a major source of IL-17. Emerging evidence indicates that IL-17 predominantly promotes cancer progression by enhancing chronic inflammation, angiogenesis, and immunosuppressive mechanisms in the tumor microenvironment, *although context-specific anti-tumor activities have been observed in some experimental models* (123). It has been shown that colorectal cancer (CRC) patients with high expression of the Th17 cells have a poor prognosis (124). A mouse model of colorectal tumorigenesis, like human colorectal cancer, exhibits upregulation of IL-23 and IL-17. It was shown that IL-23 signaling promotes tumor growth and progression, and development of a tumoral IL-17 response (125). The role of Th17 in developed cancers is limited and data is conflicting (126). On the other hand, a global population-based cohort study found that psoriasis patients who used IL-17 inhibitors were protected against eight types of cancer (127).

(b) IL-1β anti-cancer role – In a CRC animal model, IL-1 receptor ablation in myeloid cells (particularly neutrophils), resulted in bacterial invasion into tumors, heightened inflammation and aggressive CRC progression. Tumor associated bacteria are essential for driving enhanced CRC in the absence of IL-1 signaling in myeloid cells (119). As stated before, IL-1 signaling is a key regulator of the IL-17 pathway, in different cell types within the CRC microenvironment (122). Denk et al. (128) show that low IL17RA expression predicts worse prognosis in late-stage CRC. Global IL-17RA loss in mice disrupted the intestinal barrier and promoted systemic fungal invasion, but deletion restricted to epithelial cells did not increase tumor progression. Fungal antigen stimulation is required for IL-17RA signaling in myeloid cells to support protective T-cell immunity including CD8+T cells.

Treatment of advanced NSCLC patients with anti–IL-1β mAbs (canakinumab) failed to prolong PFS or OS across three Phase III clinical trials (CANOPY-1, CANOPY-2, and CANOPY-A) under different therapy settings (129–131). It can be concluded that although many preclinical studies demonstrate a pro-tumorigenic role for IL-1β, in human NSCLC its anti-tumor immune effects appear to counterbalance this activity, resulting in no clear clinical benefit from IL-1β blockade in this type of cancer.

*IL-6*: In a chemical carcinogenesis model, IL-6 knockout (IL-6^-^/^-^) mice developed fewer gastric tumors compared with wild-type mice. Tumor development was associated with STAT3 activation, which was reduced in the absence of IL-6 (132). However, in a genetically engineered mouse model of lung cancer driven by an activated K−Ras oncogene, IL−6 deficiency actually led to increased tumor initiation compared with wild−type mice, suggesting that IL−6 has a dual role — it can suppress early tumor formation through immune regulation and homeostasis but then promote later tumor growth via STAT3 signaling (133). Aberrant activation of the IL-6/JAK/STAT3 pathway is common across many types of cancer and is generally associated with poor prognosis. Within the tumor microenvironment, this signaling axis promotes tumor cell proliferation, survival, invasion, and metastasis, while concomitantly suppressing antitumor immune responses. Accordingly, therapeutic targeting of the IL-6/JAK/STAT3 pathway has been proposed to confer clinical benefit by both restraining tumor growth and enhancing antitumor immunity (134, 135). Currently, no anti-IL-6 agent is approved for the treatment of cancer. Although siltuximab is approved for multicentric Castleman disease, this lymphoproliferative disorder is not classified as a malignancy. Despite a strong preclinical rationale and evidence of IL-6 pathway involvement across multiple tumor types, large RCTs evaluating anti-IL-6 therapies in oncology have generally failed to demonstrate a clear survival benefit (136, 137). Similarly, JAK inhibition has not demonstrated consistent efficacy or survival benefit in most clinical studies of solid tumors, with the notable exception of a trial reporting improved overall survival in a subgroup of patients with pancreatic ductal adenocarcinoma and systemic inflammation treated with ruxolitinib (138). To date, no JAK inhibitor is approved for the treatment of non-hematologic malignancies, although several are approved for hematologic disorders, including myelofibrosis (e.g., ruxolitinib and fedratinib) and polycythemia vera (ruxolitinib).

Regarding cancer incidence, a Japanese study found that the rate of malignancies among patients receiving tocilizumab was comparable to that observed in an observational cohort of patients with rheumatoid arthritis and in the general Japanese population (139, 140).

*TGF-β1*: Neutrophilic asthma is associated with higher TGF-β levels than allergic rhinitis (AR). In cancer types in which the TGF-β/Smad pathway is functionally intact, cancer risk is higher in neutrophilic asthma than in AR, whereas in cancers with impaired TGF-β/Smad signaling (e.g., due to high local sex hormones), neutrophilic asthma is not associated with excess risk (10). These data suggest that TGF-β signaling contributes to cancer onset. However, multiple preclinical and genetic studies indicate that loss of TGF-β signaling increases cancer susceptibility. Mice with defective TGF-β signaling or loss of TGF-βRII are more susceptible to chemically induced CRC than WT mice (141, 142). In humans, juvenile polyposis—caused in 40–60% of cases by SMAD4 or BMPR1A mutations (143)—is associated with a 34% relative risk of CRC (144).

This apparent discrepancy can be explained by the pleiotropic, concentration-dependent effects of TGF-β. At low levels, TGF-β promotes Th17 differentiation (together with IL-6, IL-21, IL-1β, or IL-23) and sustains a pro-inflammatory milieu (85). TGF-β regulates Th17 development in a dose-dependent manner: low concentrations enhance IL-23R expression and Th17 differentiation, whereas high concentrations repress IL-23R and favor Foxp3^+^ Treg differentiation (145). Thus, the relationship between TGF-β and Th17 differentiation follows a bell-shaped curve. Accordingly, TGF-β levels in neutrophilic asthma may fall within the pro-Th17, pro-inflammatory range, increasing cancer initiation risk. In contrast, if basal TGF-β levels in WT mice lie above this peak and favor Tregs, then partial loss of TGF-β signaling would increase CRC susceptibility, as observed experimentally (141). In early cancer, moderately elevated TGF-β suppresses epithelial proliferation. In advanced cancer, however, TGF-β signaling is frequently disrupted by SMAD4 or TGF-βRII mutations or deletions (CRC (146), pancreatic ductal denocarcinoma (147), gastric cancer (148), breast cancer (149)), rendering tumor cells resistant to its suppressive effects. At the same time, high TGF-β levels in the TME—secreted by Tregs and cancer cells—suppress antitumor immunity and promote tumor progression (150).

Collectively, TGF-β plays three distinct roles in cancer: (1) at very low levels it promotes Th17 differentiation and a pro-inflammatory environment that drives cancer onset; (2) at higher levels it suppresses early tumor development through its cytostatic effects; and (3) once tumor cells acquire resistance to TGF-β–mediated growth inhibition, persistently high TGF-β promotes tumor progression by inducing immune suppression and a permissive tumor microenvironment.

These data are presented in **Table 2**.

**Table 2:** Biological and clinical effects of IL-1β, IL-6, and TGF-β1 in different diseases and health conditions.

Disease/condition	IL-1β	IL-6	TGF-β1
Metabolic Syndrome	Chronic IL-1β exposure induces IR, whereas short exposure stimulates insulin secretion (29, 30). IL-1β induces IL-6 secretion (31). Anakinra and canakinumab (inhibitors of the IL-1 pathway) produced only modest effects on T2D hyperglycemia (33, 34).	Elevated serum levels of IL-6 were associated with hypertriglyceridemia and insulin resistance (49), and aqueous humor levels correlated with the severity of diabetic retinopathy (42). Conflicting data exist on serum IL-6 levels in PCOS compared with controls (45), but elevated IL-6 is reported in obese PCOS patients (46). Tocilizumab (anti–IL-6 mAb) improved IR in RA patients (52, 53). IL-6 pathway inhibitors reduce HbA1c (54, 55) but induced weight gain (50).	The TGF-β/Smad3 pathway plays a key role in obesity and pancreatic β-cell dysfunction (56–59).
MASH	IL-1β drives hepatic inflammation and fibrosis (63) and stimulates hepatocyte-mediated neutrophil recruitment and activation (64).	Animal models show conflicting roles (protective vs detrimental) (67–72).	TGF-β1/Smad3 signaling is a major driver of liver fibrosis via hepatic stellate cell activation (77).
Neutrophilic Asthma	The inflammasome proteins NLRP, NLRP3 and NLRC4 are associated with neutrophilic asthma and with sputum IL-1β protein levels (151).	Serum IL-6 and sIL-6R levels were positively correlated with blood neutrophil counts in severe asthma (152)	TGF-β, together with IL-1β and IL-6 (± IL-21/IL-23), promotes Th17 differentiation (85). Th17 cells recruit neutrophils that further release TGF-β1 (88).
Osteoarthritis	IL-1β is a major inflammatory driver of OA (89).	IL-6 is a major inflammatory driver of OA (89); elevated serum IL-6 is associated with knee OA and cartilage loss (97).	Chondrocytes activate two distinct TGF-β1 pathways: one protective and one degradative; thus, TGF-β1 has pleiotropic effects in OA (98).
Cardiovascular pathology	IL-1β independently predicts CVD risk in drug-naïve T2D (103). Canakinumab (150 mg q3mo) reduced recurrent CV events independent of lipid lowering (104).	IL-6 promotes the development of atherosclerosis, coronary artery disease, hypertension, arterial aneurysm, aortic dissection, cardiac fibrosis, myocarditis, and cardiac ischemia-reperfusion injury (106). Anti-IL-6 treatment effectively reduced atherosclerosis (107, 108)	Dysregulated TGF-β signaling is a hallmark of vascular disease (109, 110). Cardiac and vascular fibrosis are linked to TGF-β signaling in preclinical and clinical studies (111–117)
Cancer onset	IL-1β induces nitric oxide and ROS, triggering tumor initiation (118, 119). IL-1β inhibition reduced lung cancer risk in atherosclerosis patients (120).	Preclinical data are conflicting and cancer-type dependent (132, 133).	At very low levels, TGF-β1 promotes Th17 differentiation and a pro-inflammatory environment driving cancer initiation (10).
Cancer progression	IL-1β drives tumor growth and metastasis across cancers (121). Conversely, IL-1R1 ablation promoted aggressive CRC (119). IL-17RA signaling in myeloid cells (driven by IL-1) supports anti-tumor T-cell immunity in CRC (128). Canakinumab did not prolong PFS or OS in NSCLC (129–131).	Despite strong preclinical rationale, IL-6 targeting has not improved survival in large oncology trials (136, 137).	TGF-β1 suppresses early tumors via cytostasis; once resistance develops, it promotes immune suppression and tumor progression (141–144, 146, 152).

CRC, colorectal cancer; CV, cardiovascular; CVD, cardiovascular disease; IR, insulin resistance; LDL, low-density lipoproteins; mAb, monoclonal antibodies; NSCLC, non–small cell lung cancer; OA, osteoarthritis; OS, overall survival; PCOS, polycystic ovary syndrome; PFS, progression-free survival; ROS, reactive oxygen species; T2D, type 2 diabetes.

## Clinical efficacy of GLP-1 RAs in managing and reducing risk of different diseases and health conditions

Metabolic syndrome: A recent meta-analysis of 47 RCTs has shown significant weight, BMI, and waist circumference reduction in patients with obesity and overweight using GLP-1 RAs (153). A systematic review of published studies showed that, after a 28.86 weeks mean treatment duration of NAFLD (now MAFLD) patients, GLP-1 receptor agonists were associated with statistically significant reductions in insulin resistance (HOMA-IR), visceral fat, body weight, fasting blood glucose, and triglyceride levels, compared with control treatments (placebo, standard care, or other antidiabetic drugs) (154).

MASH: A recent meta-analysis of 13 RCTs concluded that, regardless of diabetes status, in individuals with MASH and moderate-to-advanced fibrosis, GLP-1RAs administered for up to 72 weeks were superior to placebo in achieving MASH resolution and improving liver fibrosis and liver fat content (155).

Asthma: Kaplan et al. (8) analyzed individuals with asthma and obesity using a database that included over 10,000 GLP-1 RA–exposed patients and more than 50,000 unexposed controls. The exposed cohort had a higher BMI and more uncontrolled asthma at baseline but experienced greater weight-loss and improved asthma control during follow-up. Using data from the global TriNetX research network, Patel et al. (9) conducted a cohort study of overweight, obese, and morbidly obese non-diabetic patients with asthma, comparing asthma exacerbation rates over a 3-year period between individuals initiating a GLP-1 receptor agonist and matched controls without GLP-1 RA exposure. Across all three BMI groups, GLP-1 RA initiation was associated with a statistically significant reduction in the risk of asthma exacerbation, with a similar risk ratio of approximately 0.78 in each group.

*Osteoarthritis:* A large retrospective analysis evaluated GLP-1 RAs use in patients without (n = 1,092,225) and with (n = 237,043) preexisting hip and/or knee osteoarthritis. Among patients with established OA, GLP-1 RAs use was associated with lower odds of progression to total hip or knee arthroplasty. In contrast, among patients without prior OA, GLP-1 RA use was associated with a higher incidence of hip and knee OA, increased use of major joint injections, and a greater likelihood of total knee arthroplasty (156).

Cardiovascular pathology: A recent meta-analysis including 21 trials encompassing almost 100,00 patients across diverse populations (diabetes mellitus, kidney function, obesity, or heart failure) found conclusive, high-certainty evidence that GLP-1 RAs reduced all-cause death, CV death, and major adverse cardiovascular events, compared with placebo or controls (157).

Cancer onset: In a cohort of 1,221,218 drug-naive patients with T2D, GLP-1 RAs were associated with a lower CRC risk than insulin (HR = 0.56), metformin (HR = 0.75), SGLT2 inhibitors, sulfonylureas, and thiazolidinediones, with nonsignificant reductions versus α-glucosidase and DPP-4 inhibitors (158). A recent review summarized multiple meta-analyses evaluating cancer risk associated with GLP-1 receptor agonist use in patients with T2D or obesity, including the large meta-analysis by Figlioli et al. (159). Several analyses reported reduced risks of hepatocellular, colorectal, prostate, and hematological cancers compared with insulin, other antihyperglycemic agents, or placebo. Figlioli et al. (160) found no significant association between GLP-1 RAs and overall or site-specific gastrointestinal cancer risk, a finding consistent across placebo-controlled, high-dose, and long-term (≥5 years) analyses. Most analyses reported no effect on pancreatic cancer risk, although one retrospective study suggested a reduced risk. Evidence regarding thyroid cancer risk was inconsistent, with some studies reporting increased risk and others no association. Two large, more recent studies estimating the risk of incident thyroid cancer among adults with T2D reported conflicting results: one found an increased risk (161), whereas the other did not (162).

In a retrospective cohort study using a target trial emulation design based on electronic health records from a large US database, 43,317 individuals with obesity treated with GLP-1 RAs were compared with 43,315 matched nonusers. GLP-1 RAs use was significantly associated with a reduced risk of overall cancer and with lower risks of endometrial (HR = 0.75), ovarian (HR = 0.53), and meningioma (HR = 0.69). For other cancer types (except kidney cancer), the estimated risks were lower, but the decreases were not statistically significant (163).

Taken together, although clinical data on the effects of GLP-1 RAs on cancer risk are conflicting, the overall evidence suggests that they reduce or do not increase the risk of most cancer types, with a possible exception for thyroid cancer.

Cancer progression: It has been reported that GLP-1 RA use was associated with improved recurrence-free survival in overweight and obese patients with NSCLC after lobectomy (HR = 0.41, P = 0.026) compared with non-users (164). Similarly, a recent global retrospective cohort study found that among diabetic patients treated for CRC, GLP-1 RA users had significantly better overall survival and metastasis-free survival than non-users [Dosda].

In a recent analysis of database that included 6871 colon-cancer patients, GLP-1 RAs use was consistently associated with a substantial reduction in five-year mortality across multiple logistic regression models, including full adjustment for BMI, disease severity, demographics, and comorbidities. However, time-to-event analyses did not demonstrate statistically significant differences in mortality hazards, suggesting that the observed benefit may reflect differences in cumulative survival rather than uniform risk reduction over time (166).

## Clinical efficacy of metformin in managing and reducing risk of different diseases and health conditions

Metabolic syndrome: Clinical studies have shown that metformin produces long-term but modest weight loss of approximately 2–4 kg in T2D patients and in healthy individuals (167) and in patients with NAFLD (168). Metformin also restores physiological insulin secretion and reduces insulin resistance in hyperinsulinemic, normoglycemic subjects with metabolic syndrome (169). Consistent with these findings, metformin improved insulin resistance in overweight and obese Mexican Mestizo subjects (170) and enhanced peripheral—but not hepatic—insulin sensitivity in obese patients with T2D (171). The beneficial effect of metformin on insulin resistance in patients with metabolic syndrome is further supported by multiple studies (172–175) and by a meta-analysis (176).

MASH:A recent review presents contrasting results regarding the effect of metformin on liver transaminases (ALT and ALT) and hepatic fat content. Although 15 studies and 4 meta-analyses demonstrate beneficial effect, 6 studies and 2 meta-analyses show that metformin treatment does not show significant improvements in ALT and AST levels or hepatic fat content. In particular, metformin is probably not effective in improving liver function in pediatric and adolescent populations (177).

Asthma: In a UK cohort of 12,702 patients with asthma, metformin use was associated with an approximately 30% lower risk of exacerbations, with a further ~40% reduction observed among those receiving concomitant GLP-1 receptor agonists, irrespective of glycemic control, body weight, or asthma phenotype (178). A smaller U.S. study of 464 patients similarly reported reduced asthma exacerbation rates following metformin initiation (179). Consistent with these findings, another U.S.-based study of 1,749 adults with diabetes showed that metformin use—independent of glycemic control and obesity—was associated with a lower hazard of asthma-related emergency department visits and hospitalizations (180). However, a meta-analysis of seven studies including 1,176,398 patients found that metformin use in type 2 diabetes was not significantly associated with reduced asthma incidence or most asthma-related outcomes, although a lower risk of asthma-related hospitalization was observed (181). Similarly, another meta-analysis including 317,905 patients with type 2 diabetes reported that metformin therapy does not significantly affect asthma outcomes (182).

Osteoarthritis: Six months of metformin treatment (2,000 mg/day) significantly improved knee pain in overweight or obese patients with knee osteoarthritis compared with placebo (183). Consistent with this finding, another study reported that metformin use may confer long-term benefits on knee joint outcomes in individuals with knee osteoarthritis and obesity (184). Furthermore, a nationwide, retrospective matched-cohort study in Taiwan found that patients with osteoarthritis and type 2 diabetes who received combination therapy with COX-2 inhibitors and metformin had lower rates of joint replacement surgery than matched controls treated with COX-2 inhibitors alone (185). However, metformin does not appear to reduce the risk of developing OA in patients with T2D. In a UK cohort of 3,217 patients with type 2 diabetes without prior osteoarthritis who were followed for approximately 8 years, metformin use—either at baseline or as a time-varying exposure—was not associated with incident OA (186).

Cardiovascular pathologies: Many preclinical and clinical trials demonstrate the protective effect of metformin in CVD as presented by an excellent review of Bu et al. (187).

Cancer onset: A meta-analysis of 37 studies comprising 1,535,636 participants, found statistically significant reduction of overall-cancer incidence (27%) and of liver (78%), pancreatic (46%), colorectal (23%) and breast cancer (6%) incidence in metformin users compared to non-users (188). In addition, two recent systematic reviews and meta-analyses found that metformin use in patients with diabetes was associated with a reduced risk of multiple cancer types; however, substantial study heterogeneity and potential publication bias limit confidence in these findings (189, 190).

Cancer progression: A meta-analysis of 20 studies including 13,008 patients with concurrent cancer and T2D reported a significant survival benefit associated with metformin use compared with other glucose-lowering therapies, for both overall survival (HR = 0.66) and cancer-specific survival (HR = 0.62). This trend was also observed across subgroups stratified by cancer type and country, and reached statistical significance in lung, breast, and prostate cancers (191).

A meta-analysis of 22 RCTs in patients with cancer found no statistically significant differences in hazard ratios for either progression-free survival (PFS) or overall survival (OS) between the metformin and control (placebo or no treatment) groups. However, subgroup analyses suggested that metformin was associated with improved PFS in patients with reproductive system cancers, while it was associated with worse PFS in digestive system cancers (192).

**Table 3** summarizes these results.

**Table 3:** Clinical efficacy of GLP-1 RAs and metformin across diseases and health conditions.

Disease/condition	GLP-1 RAs effect	Metformin effect
Metabolic syndrome	Significant weight, BMI, and waist circumference reduction in obese patients (153). Significant reductions in IR, visceral fat, body weight, fasting blood glucose, and triglyceride levels in patients with MAFLD (154).	Modest weight loss; improves IR in high-risk T2D/CVD and various obese populations; reduces fasting insulin, HOMA, waist, and testosterone in women with PCOS; improves peripheral (but not hepatic) IR in obese T2D patients (167–175)
MASH	Improvement in MASH resolution, liver fibrosis, and liver fat content (155)	Contrasting results (177)
Asthma	Improve asthma control in people with obesity (8), and in overweight, obese, and morbidly obese non-diabetic patients (9).	Contrasting results (178–182)
Osteoarthritis	Improves symptoms in established OA but increases OA risk in those without prior OA (156).	Contrasting results (183–186)
Cardiovascular pathology	Reduced all-cause death, CV death, and MACE (157).	Reduced all-cause death, CV death, and MACE (187).
Cancer onset	Reduced or no increased risks in most cancers with possible exception of thyroid cancer (158–160, 163).	Reduced risk of multiple cancers (188–190).
Cancer progression	Prolonged survival in lung and colon cancer patients (164, 165). Lower 5-year mortality, but no significant effect in time-to-event analyses in CRC (166).	Survival benefit across cancers and in lung, breast, and prostate cancers versus other glucose-lowering therapies (191). No significant differences in PFS or OS overall; improved PFS in reproductive system cancers but worse PFS in digestive system cancers (192).

BMI, body mass index; CV, cardiovascular; CVD, cardiovascular disease; CRC, colorectal cancer; HOMA, homeostatic model assessment; IR, insulin resistance; MACE, major adverse cardiovascular events; MAFLD, metabolic associated fatty liver disease; OA, osteoarthritis; OS, overall survival, PFS, progression-free survival.

## Comparative efficacy of GLP-1 receptor agonists and metformin across diseases and health conditions

HbA1c (in T2D patients): A retrospective study using Danish nationwide registers data, identified 1778 Danish drug-naïve pre-diabetic and diabetic patients who started to use GLP-1 RAs or metformin from 2018 to 2021. GLP-1 RA use was associated with statistically significant greater HbA1c reduction compared to metformin (the difference was 7%-10%) (193).

Insulin resistance (in PCOS patients): A meta-analysis of seven RCTs comprising 464 overweight/obese women with PCOS found that GLP-1 RAs appear to be more beneficial for insulin resistance (and weight loss) improvement compared to metformin (194).

Weight loss: In a Phase 3, placebo-controlled clinical trial evaluating Wegovy^®^ (semaglutide) in obese or overweight adults with at least one weight-related comorbid condition (e.g., treated or untreated dyslipidemia or hypertension, but without type 2 diabetes), mean body weight decreased by 14.9% from baseline after 68 weeks of therapy. In another Phase 3, placebo-controlled clinical trial evaluating Wegovy^®^ (semaglutide) in obese or overweight adults with T2D, mean body weight decreased by 9.6% from baseline after 68 weeks of therapy (4). By contrast, metformin has been shown to induce modest long-term weight loss of about 2–4 kg (2.7%-5.5%)in patients with type 2 diabetes and in healthy subjects (167). A study that compared directly GLP-1 RAs with metformin has demonstrated a better efficacy of GLP-1 RAs in reducing body weight in patients with concomitant T2D and NAFLD (168).

MASH: In comparative studies of patients with NAFLD and concomitant T2D, treatment with GLP-1 receptor agonists demonstrated greater improvements in liver function than treatment with metformin (168, 195).

Polycystic ovary syndrome: A meta-analysis comparing the efficacy and safety of GLP-1 receptor agonists in women with PCOS with these of metformin, concluded that compared with metformin, GLP-1 receptor agonists were more effective in improving insulin sensitivity and reducing body mass index and abdominal girth. However, GLP-1 receptor agonists were associated with a higher incidence of nausea and headache than metformin (196).

Asthma: Kimura et al. (197) conducted a new-user cohort study using a Japanese national administrative database, comparing antihyperglycemic drugs with metformin in 137,173 patients with a history of asthma who initiated therapy between 2014 and 2022. They concluded that GLP-1 RAs were associated with poorer control of asthma compared with metformin. However, a meta-analysis of six clinical studies involving metformin reported that the study by Kimura et al. was at moderate risk of bias (182).

Osteoarthritis: A comparative meta-analysis of antidiabetic drugs in obese patients with knee osteoarthritis found that metformin was superior to semaglutide in relieving knee pain (198). In contrast, preoperative use of GLP-1 receptor agonists was superior to metformin use in reducing the 90-day risk of periprosthetic joint infection (RR = 0.58 vs 0.77) and hospital readmission (RR = 0.53 vs 0.92), based on two retrospective cohort studies (199, 200).

Cardiovascular Diseases: As mentioned above, a recent meta-analysis of 21 trials across diverse populations found high-certainty evidence that GLP-1 receptor agonists reduce all-cause mortality, cardiovascular mortality, and MACE compared with placebo or control treatments (157). In contrast, a meta-analysis of 29 studies investigating the association of metformin use with CV risk in patients with T2D, concluded that metformin treatment was not associated with reduced risk of all-cause mortality, cardiovascular mortality, macrovascular events, heart failure and microvascular events (201).

Cancer onset: In a U.S. nationwide multicenter retrospective cohort of over 1.5 million patients with T2D without prior obesity-associated cancers (2005–2018), treatment with GLP-1 receptor agonists was not associated with a lower overall cancer risk compared with metformin, but was linked to an increased risk of kidney cancer (202).

Cancer progression: Analysis of a large global database identified a cohort of 3747 cancer patients with T2D who received GLP-1 RAs within 3 months of starting systemic therapy which was compared to a cohort of 52,061 cancer patients with T2D receiving metformin in the same timeframe. It was found that patients with diabetes and cancer who received GLP-1 RAs experienced superior survival outcomes and reduced rates of hospitalization compared to patients receiving metformin (203).

## GLP-1 RA–induced weight loss is mediated by central nervous system and peripheral mechanisms independent of systemic IL-6 levels

In a randomized placebo-controlled trial involving 30 individuals with obesity, Blundell et al. (204) showed that once-weekly semaglutide (escalated to 1.0 mg) significantly reduced ad libitum energy intake and body weight compared with placebo. The weight loss was associated with reduced appetite and food cravings, improved control of eating behavior, and a lower preference for energy-dense, high-fat foods. Within the central nervous system, GLP-1 RAs act on brain regions involved in appetite regulation, modulating neurotransmitter and neuropeptide signaling to reduce hunger and influence energy balance. In peripheral tissues, they improve glycemic control by enhancing glucose-dependent insulin secretion, suppressing glucagon release, slowing gastric emptying, and modulating gut hormone signaling (205). The central effects of GLP-1 RAs are not shared by metformin. Among peripheral effects, metformin may influence gut hormone signaling (206), but this occurs indirectly, potentially via its effects on the gut microbiome (207). Importantly, none of these effects are thought to be primarily mediated by changes in circulating IL-6.

These results are summarized in **Table 4** together with possible explanation for the reported hierarchy, which will be discussed in the discussion section.

**Table 4:** Comparative efficacy of GLP-1 RAs and metformin across diseases and health conditions.

Disease/condition	Markers	GLP-1 RAs assessed	Comparative efficacy	Possible explanation of the reported hierarchy	IL-6 involvement in the pathology
HbA1c (in T2D)	HbA1c reduction	Various	GLP-1 RAs ≥ Metformin (193)	Superior systemic IL-6 suppression by GLP-1 RAs (11, 25). Direct interference with the IL-6 pathway reduces HbA1c (54, 55)	Yes
Insulin resistance (in PCOS)	HOMA	liraglutide and exenatide	GLP-1 RAs > Metformin (194, 196)	Superior IL-6 suppression by GLP-1 RAs (11, 25). IL-6 pathway inhibition (tocilizumab) improves HOMA in rheumatoid disease (52, 53).	Yes
Obesity	Weight loss	Semaglutide, Exenatide	GLP-1 RAs > Metformin (4, 167, 168)	GLP-1 RAs suppress appetite, enhance satiety and reduce energy intake via central mechanisms (205). These primary effects on weight loss, not shared by metformin are unrelated to IL-6 signaling.	No
MASH	BMI, waist-to-hip ratio, 2-h PPG, ALT, AST, γ-GT, hs-CRP	Exenatide, Liraglutide	GLP-1 RAs > Metformin (168, 195)	Percent weight loss correlates with NASH score improvement (208). GLP-1 RAs induce greater weight loss than metformin (168) via CNS-mediated appetite suppression and delayed gastric emptying, independent of IL-6.	No
Polycystic ovary syndrome	Insulin sensitivity, BMI, abdominal girth	Various	GLP-1 RAs > Metformin (196)	Weight management is first-line therapy in PCOS (44). GLP-1 RAs produce greater weight loss than metformin (168) via appetite suppression and delayed gastric emptying, independent of IL-6.	No
Asthma	Exacerbations requiring systemic corticosteroids	Various	Metformin > GLP-1 RAs (197)	Neutrophilic asthma comprises only 20%-30% of asthma cases in adults (81, 82); IL-6 interference does not improve asthma (209). Metformin does not suppress systemic IL-6 (25).	Low involvement in general asthma; high involvement in neutrophilic asthma.
Osteoarthritis (joint pain)	Knee pain	Semaglutide, Liraglutide	Metformin > GLP-1 RAs [198 Jiang G]	IL-6 inhibition does not improve OA pain (210). Metformin does not reduce systemic IL-6 (25).	No
Osteoarthritis (perioperative)	90-day PJI and readmission	Various	GLP-1 RAs > Metformin (199, 200)	Hyperglycemia is strongly associated with periprosthetic joint infection (211). GLP-1 RAs reduce blood glucose more effectively than metformin (193), likely via superior IL-6 suppression.	Yes (indirectly)
Cardiovascular pathology	Reducing all-cause mortality, CV mortality, and MACE	Various	GLP-1 RAs > Metformin (157, 201)	Superior systemic IL-6 suppression by GLP-1 RAs (11, 25). Anti-IL-6 therapy effectively reduces atherosclerosis (107, 108)	Yes
Cancer risk	Incident cancer	Various	GLP-1 RAs ≈ Metformin for most types of cancer. GLP-1 RAs are associated with higher cancer risk compared to metformin in kidney cancer (202)	Malignancy rates in RA patients receiving tocilizumab were similar to background populations (139, 140), suggesting IL-6 interference does not drive cancer initiation.	Probably no
Cancer progression	Survival	Various	GLP-1 RAs > Metformin in cancer patients with T2D (203)	Obesity and a higher metabolic syndrome burden are associated with increased overall cancer mortality (212, 213). Appetite suppression, satiety enhancement and energy intake reduction are mechanisms associated with GLP-1 RAs (205), not shared by metformin. In addition, interference with the IL-6 pathway by GLP-1 RAs reduces HbA1c more effectively than metformin (54, 55). IL-6 targeting has not improved survival in large oncology trials (136, 137)	No (or minor)

ALT, alanine aminotransferase; AST, aspartate aminotransferase; BMI, body mass index; HbA1c, glycated hemoglobin; HOMA, homeostatic model assessment of insulin resistance; hs-CRP, high-sensitivity C-reactive protein; MACE, major adverse cardiovascular events; NASH, non-alcoholic steatohepatitis (now termed MASH); PPG, postprandial plasma glucose; γ-GT, gamma-glutamyl transferase; RA, rheumatoid arthritis.

## Differential anti-inflammatory effects of GLP-1 receptor agonists and metformin

GLP-1 RAs: The effect of semaglutide 2.4 mg once weekly on CRP in adults with overweight or obesity, with or without T2D, was evaluated in three placebo-controlled studies. Across all trials, semaglutide 2.4 mg significantly reduced CRP at week 68. The estimated treatment difference versus placebo was −44% in participants without T2D and −48% in those with T2D. When semaglutide 1.0 mg once weekly was used as a comparator, the estimated treatment difference was −39% in overweight or obese patients with T2D (214). A meta-analysis of 9 studies including 1,494 T2D patients on the effect of liraglutide on CRP reported a highly significant decrease in CRP levels. The weighted mean differences were −0.692 mg/L and -0.467 mg/L for doses > 1.6 mg/day and doses < 1.6 mg/day, respectively (215). Median plasma CRP in 112,815 Danish individuals with T2D was 2.1 mg/L (216). Because cases were accrued between 1976 and ~2015—largely predating the availability of GLP-1 receptor agonists—this cohort can reasonably be considered GLP-1RA-naïve. Using this value of 2.1 mg/L, it can be estimated that according to the meta-analysis aforementioned (215), the use of liraglutide in T2D patients is associated with an approximate 33% mean reduction in blood CRP at doses >1.6 mg/day and a 22% mean reduction at doses <1.6 mg/day.

*Metformin*: In adults with T2D with metformin dose titration targeting fasting blood glucose less than 110 mg/dL, reductions in hsCRP in patients treated with metformin (-16.1%) were not statistically different than reductions with placebo (-19.0%) (217).

A strong correlation between CRP and two IL-6 assays (r = 0.74 and r = 0.71; p < 0.001) was reported following analysis of blood samples collected from 96 patients with acute infections, autoimmune or inflammatory disease presentations (218).

It may be concluded that semaglutide 2.4 mg once weekly and daily liraglutide (at different doses) are associated with significant reductions in blood CRP and IL-6 levels compared with therapeutically effective doses of metformin.

## Gastrointestinal adverse effects are frequent with both GLP-1 RAs and metformin, whereas allergic reactions are more frequent with GLP-1 RAs: a potential role for IL-6

**Table 5** presents GI and allergic side effects of five GLP-1 RAs, metformin and four drugs that target the IL-6 signaling pathway, extracted from the drugs prescribing information in the UK (219–228).

**Table 5:** Adverse gastrointestinal events and adverse allergic reactions for 5 GPL-1 RAs, metformin and four monoclonal antibodies that target the IL-6 signaling pathway.

Drug	Adverse gastrointestinal events frequency	Adverse allergic reactions frequency
Semaglutide	very common or common	Uncommon (hypersensitivity)
Tirzepatide	very common or common	Common (hypersensitivity)
Dulaglutide	very common	Uncommon (hypersensitivity)
Liraglutide	very common or common	Uncommon (urticaria)
Exenatide (10 mcg solution)	very common or common	Common (Pruritus, and**/**or urticaria)
Metformin	Very common	Very rare (urticaria)
Siltuximab	Very common	very common (rash, pruritus, eczema) or common (anaphylactic reaction)
tocilizumab	Common or uncommon	Common (rash, pruritus, urticaria)
satralizumab	Common	Common(allergic rhinitis, rash)
sarilumab	Rare	Not known

Data is extracted from the drugs prescribing information. Frequencies are defined as follows: very common: (≥ 1/10); common (≥ 1/100 to < 1/10); uncommon (≥ 1/1,000 to < 1/100); rare (≥ 1/10,000 to < 1/1,000); very rare (< 1/10,000), frequency not known.

It can be seen that GI adverse events are frequent with GL-P1 RAs, metformin, and three of the four marketed monoclonal antibodies (mAbs) that interfere with the IL-6 pathway. Adverse allergic reactions frequencies range from common to uncommon for the five GLP-1 RAs and the three mAbs but are very rare for metformin.

Although metformin does not substantially reduce circulating IL-6 levels in patients with T2D (25), it significantly decreases IL-6 mRNA expression locally in the gastrointestinal tract in mice with induced colitis (229). The expression of IL-6 was also significantly decreased after metformin treatment in the intestine of both HFD- and LFD-fed older mice compared to their untreated controls (230). Moreover, it was found that metformin blocks the IL-6 signaling pathway in human esophageal squamous cell carcinoma cell line (231). In addition, researchers derived a gene signature from metformin-treated colon cancer cells and applied it to The Cancer Genome Atlas colon adenocarcinoma data to identify patients with similar expression profiles. This patient group was characterized by a better prognosis and decreased IL-6 pathway signaling (232). Metformin has also been shown to downregulate IL-6 signaling in other human epithelial tissues such as ovarian cancer cells (233). It seems that local IL-6 suppression may help explain why metformin, similar to GLP-1 RAs and several IL-6 pathway inhibitors, is frequently associated with GI AEs.

The reduction in IL-6 expression observed in the colon tissue of metformin-treated IBD mice (229), in the intestines of high-fat and low-fat diet older mice (230), and in human esophageal squamous cell carcinoma (SCC) cell lines (231) may be attributable to the high local concentrations of metformin used in these models. In the SCC model, the decrease in IL-6 expression was associated with downregulation of the IL-6/JAK2/STAT3 signaling pathway (231), and may similarly explain the reduction in phosphorylated STAT3 observed in the IBD model (229), thereby contributing to local anti-inflammatory effects.

Metformin concentrations used in these studies ranged from 0.2–1 mM (229) and 5–10 mM (231), corresponding to approximately 25,800–129,000 ng/mL and 646,000–1,292,000 ng/mL, respectively. In contrast, steady-state plasma concentrations following once-daily administration of 1000 mg metformin in healthy subjects are typically in the range of hundreds to a few thousand ng/mL (234), approximately 25- to 1300-fold lower than those used in the experimental models. Notably, the effect of metformin on IL-6 expression in SCC cells was dose-dependent (231) (this was not assessed in the IBD model). Taken together, differences in metformin exposure may account for the discrepancy between findings in experimental models evaluating the effect of metformin on cell lines or colon tissues, and clinical studies evaluating the effect of metformin on circulating IL-6. However, because metformin concentrations in the gastrointestinal lumen following oral administration are expected to greatly exceed plasma concentrations, local effects in the human gut may more closely resemble those observed in these experimental models. Suppressed IL-6 levels in the GI tract may further explain the GI adverse effects commonly associated with metformin use.

Defective IL-6 signaling, caused by mutations in the IL-6 receptor complex or downstream mediators such as STAT3, leads to pathological Th2-biased immune responses (235). Accordingly, GLP-1 RAs and direct inhibitors of the IL-6 signaling pathway are more frequently associated with allergic reactions than metformin, which exerts minimal systemic interference with this pathway and only rarely induces allergic reactions.

## Discussion

As shown in **Table 5**, GLP-1 receptor agonists demonstrate superior efficacy compared with metformin in the treatment of hyperglycemia, insulin resistance (in PCOS), obesity, MASH, polycystic ovary syndrome, cardiovascular pathology, and in reducing the 90-day risk of periprosthetic joint infection and hospital readmission in patients with osteoarthritis. In contrast, metformin is more effective in the treatment of asthma and osteoarthritis-associated joint pain.

**Figure 1:**
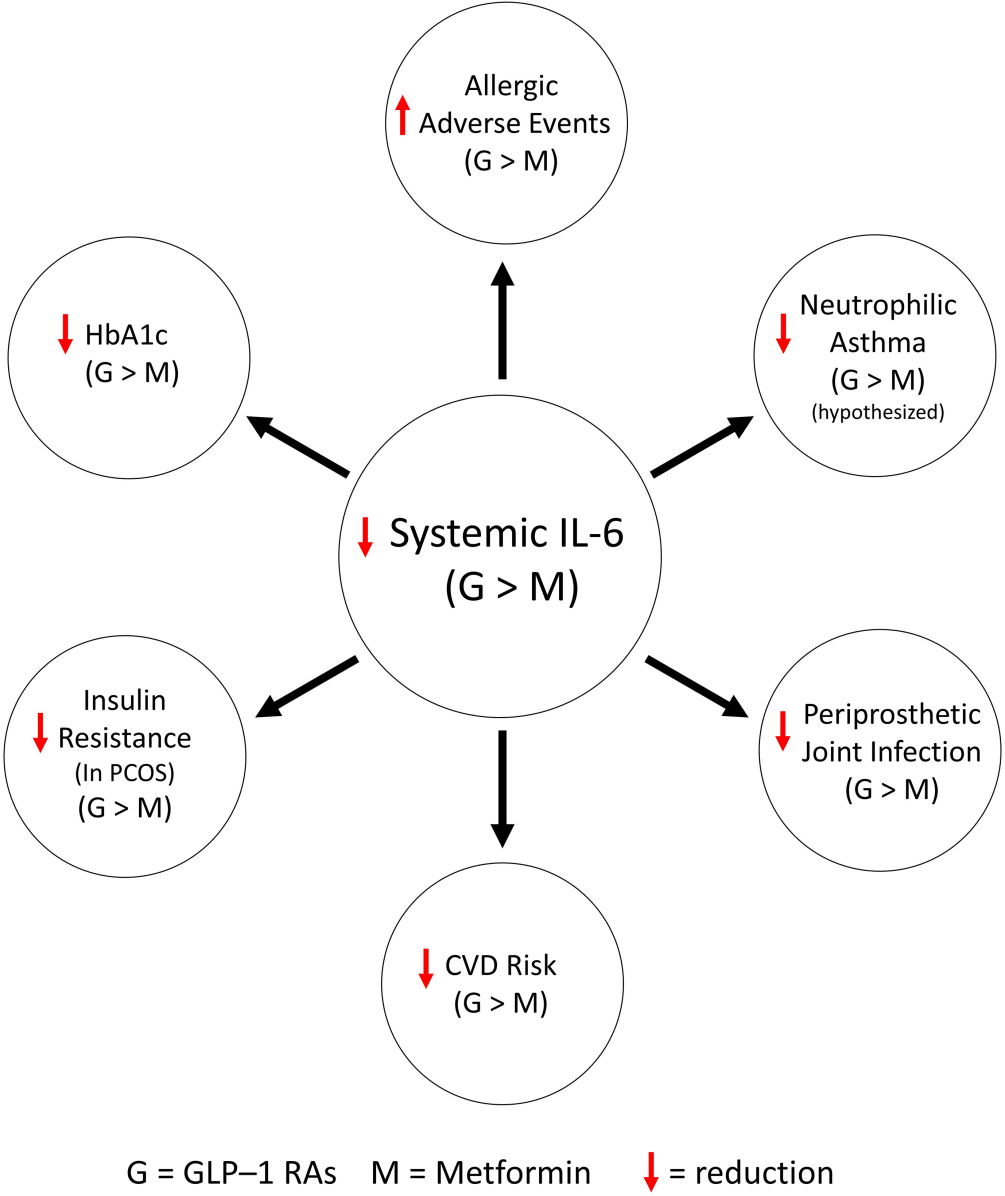
Effects of systemic IL-6 reduction across different health conditions. GLP-1 RAs reduce circulating IL-1β, IL-6, and TGF-β1, whereas metformin lowers IL-1β and TGF-β1 but has little effect on IL-6. This difference likely contributes to the greater efficacy of GLP-1 RAs in reducing HbA1c, insulin resistance (in PCOS), periprosthetic joint infection, and cardiovascular risk, while also accounting for the increased rate of allergic adverse events compared with metformin, as IL-6 pathway inhibitors have been shown to reduce HbA1c, insulin resistance, and atherosclerotic event risk and are frequently associated with hypersensitivity reactions. It is further hypothesized that GLP-1 RAs may be superior to metformin in alleviating neutrophilic asthma, given the involvement of IL-6 in the pathophysiology of this asthma subtype.

GLP-1 RAs significantly reduce circulating IL-6 levels in patients with T2D (11), whereas metformin does not lower systemic IL-6 (25). Consistently, GLP-1 RAs produce a substantially greater reduction in CRP (214, 217), and consequently IL-6 (218), than metformin. It is therefore proposed that the stronger effect of GLP-1 RAs on lowering systemic IL-6 contributes to their superior efficacy in improving hyperglycemia, insulin resistance (in PCOS), cardiovascular events, and periprosthetic joint infection risk (**Figure 1**). In all these conditions, blockade of the IL-6 pathway has demonstrated beneficial effects (52–55, 107, 108). However, there is no evidence that interference with IL-6 signaling is effective in reducing weight or in improving MASH and PCOS. Moreover, IL-6 pathway inhibition has not been shown to improve asthma [209 Revez], osteoarthritis-related joint pain (210), or cancer survival (136, 137), nor to reduce the risk of malignancy (139, 140).

**Figure 2:**
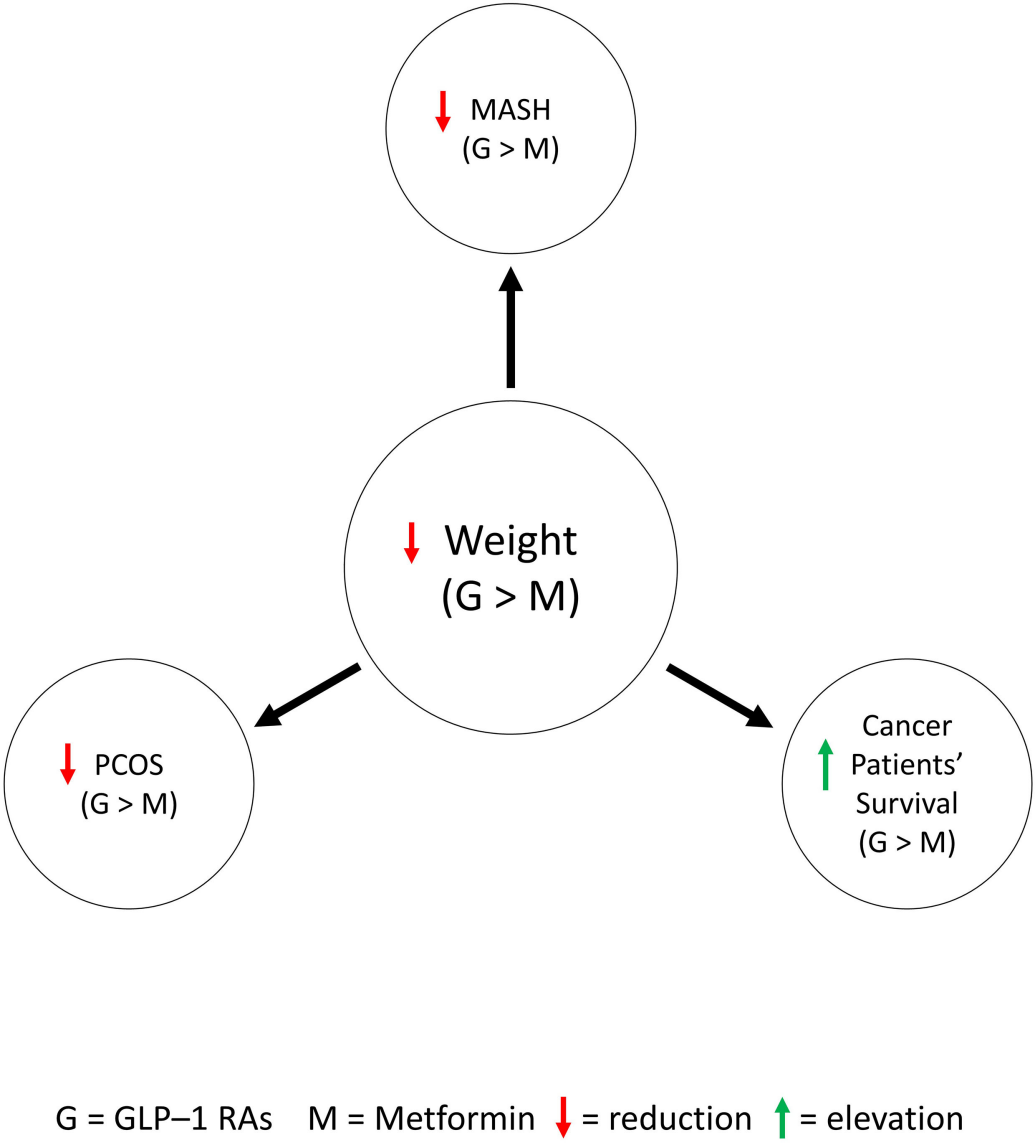
Effect of weight loss across different health conditions. The greater weight loss induced by GLP-1 RAs compared with metformin accounts for their superior efficacy in treating metabolic dysfunction–associated steatohepatitis (MASH) and polycystic ovary syndrome (PCOS), two conditions in which weight control is a key therapeutic target. In addition, because obesity is associated with increased cancer mortality, GLP-1 RAs prolong survival in cancer patients to a greater extent than metformin. These superior effects of GLP-1 RAs in these conditions are independent of the IL-6 signaling pathway.

In contrast, weight reduction improves MASH (208), PCOS (44), and prolongs survival in cancer patients (212, 213) (**Figure 2**). The greater efficacy of GLP-1 RAs in inducing weight loss is likely unrelated to their stronger effects on the IL-6 signaling pathway, as inhibition of this pathway has been shown to increase weight and BMI, particularly in patients with rheumatoid arthritis (50). The central nervous system actions of GLP-1 RAs—namely reductions in appetite, hunger, and energy intake—are the primary drivers of GLP-1 RA–associated weight loss (204, 205). These effects are not shared by metformin and occur independently of IL-6 signaling. Collectively, weight reduction—rather than IL-6 pathway suppression—likely explains the superiority of GLP-1 RAs over metformin in improving MASH and PCOS and in prolonging survival in cancer.

As noted above, although GLP-1 RAs are more effective than metformin in the management of most conditions listed in **Table 5**, the opposite is observed for asthma and osteoarthritis-related knee pain. If the superior efficacy of GLP-1 RAs in alleviating these two conditions is driven primarily by stronger suppression of the IL-6 signaling pathway, then IL-6 signaling would be expected to play a major pathogenic role in these two disorders. However, a clinical trial in 66 patients with mild asthma demonstrated that a single dose of tocilizumab, an anti–IL-6 receptor antibody, failed to prevent allergen-induced bronchoconstriction (209). Similarly, in a multicenter, 12-week, randomized, double-blind, placebo-controlled study involving 104 patients with hand osteoarthritis, tocilizumab was no more effective than placebo for pain relief (210). Asthma comprises two major inflammatory endotypes: neutrophilic asthma, driven by a cytokine network that includes IL-6, IL-1β, and TGF-β1, and eosinophilic asthma, mediated by IL-4, IL-5, and IL-13 (10). Because 70%-80% of adult patients with asthma do not exhibit a neutrophilic phenotype (81, 82), the contribution of IL-6 is likely less prominent in the general asthma population. This may explain the failure of tocilizumab to improve asthma in a clinical trial (209). However, the present analysis proposes that tocilizumab (and other IL-6 pathway inhibitors) may be effective in patients with the neutrophilic asthma phenotype. The opposite effect may be observed in patients with neutrophilic asthma.

With respect to cancer onset and early growth, chronic inflammation can induce genetic alterations that activate oncogenes or inactivate tumor-suppressor genes, thereby facilitating tumor initiation (236). In humans, combinations of IL-1β and IL-6 (237), IL-1β and IL-23 (238), or low levels of TGF-β1 together with IL-6, IL-21, or IL-23 (85, 145) drive Th17 differentiation and sustain a pro-inflammatory environment. At the same time, TGF-β1 suppresses tumor growth at early stages (239), before mutations or deletions in SMAD4 or TGF-βRII disrupt TGF-β signaling (146–149). Although GLP-1 RAs and metformin generally reduce, or do not increase, the risk of most cancer types, the balance between the pro-tumor and anti-tumor effects induced by each drug may underlie their differential effects across cancer types. While both drugs have been associated with reduced cancer risk, the magnitude and statistical significance of these effects differ considerably across cancer types. Dai et al. (163) found that GLP-1 receptor agonist use in obese individuals was significantly associated with a lower risk of endometrial and ovarian cancers, whereas risk reductions for most other cancer types were not statistically significant. Consistent with this finding, estrogens and progesterone have been reported to inhibit TGF-β signaling through suppression of Smad2/3 phosphorylation (240, 241). Estrogen concentrations in ovarian tissue are at least 100-fold higher than circulating levels (242). Many established risk factors for endometrial cancer involve excess estrogen exposure or enhanced estrogen signaling, and most endometrial tumors express high levels of estrogen receptor-α (243). Impaired TGF-β signaling in female reproductive tissues may therefore explain the more pronounced reductions in endometrial and ovarian cancer risk reported by Dai et al. (163) compared with other cancer types. Similarly, sex hormones play a central role in the pathogenesis of meningiomas, as reflected by their higher incidence in women, accelerated growth during pregnancy, increased risk in patients with breast cancer, expression of hormone receptors in tumor tissue, and the elevated risk observed in men treated with anti-androgens (244). Consistent with this pattern, meningioma—together with endometrial and ovarian cancers—was among the few cancer types associated with a statistically significant reduction in risk following GLP-1 RA use (163).

A meta-analysis of 203 studies including 6,320,365 patients with cancer found that obesity was associated with higher overall cancer mortality. However, in lung cancer, renal cell carcinoma, and melanoma, patients with obesity had a lower risk of death than patients with the same cancers without obesity, consistent with the so-called “obesity paradox” (212).

Using data from a Catalonian population-based cohort, remaining life expectancy across multiple cancer types was inversely associated with the number of metabolic syndrome components. A higher metabolic syndrome burden was linked to reduced remaining life expectancy in at least eight cancer types in men and nine in women (213).

Given the superior efficacy of GLP-1 receptor agonists compared with metformin in reducing HbA1c and inducing weight loss (**Table 5**), these metabolic benefits may contribute to the greater survival advantage observed with GLP-1 RA use across multiple cancer types. This conclusion is supported by two studies: Xie et al. (245) reported that HbA1c was associated with all-cause mortality in cancer survivors, while Mahadevan et al. (203) found that GLP-1 RA use in obese patients was associated with improved survival that approached statistical significance (HR = 0.877; P = 0.156) compared with metformin, whereas survival was similar between treatments in non-obese patients.

**Figure 3:**
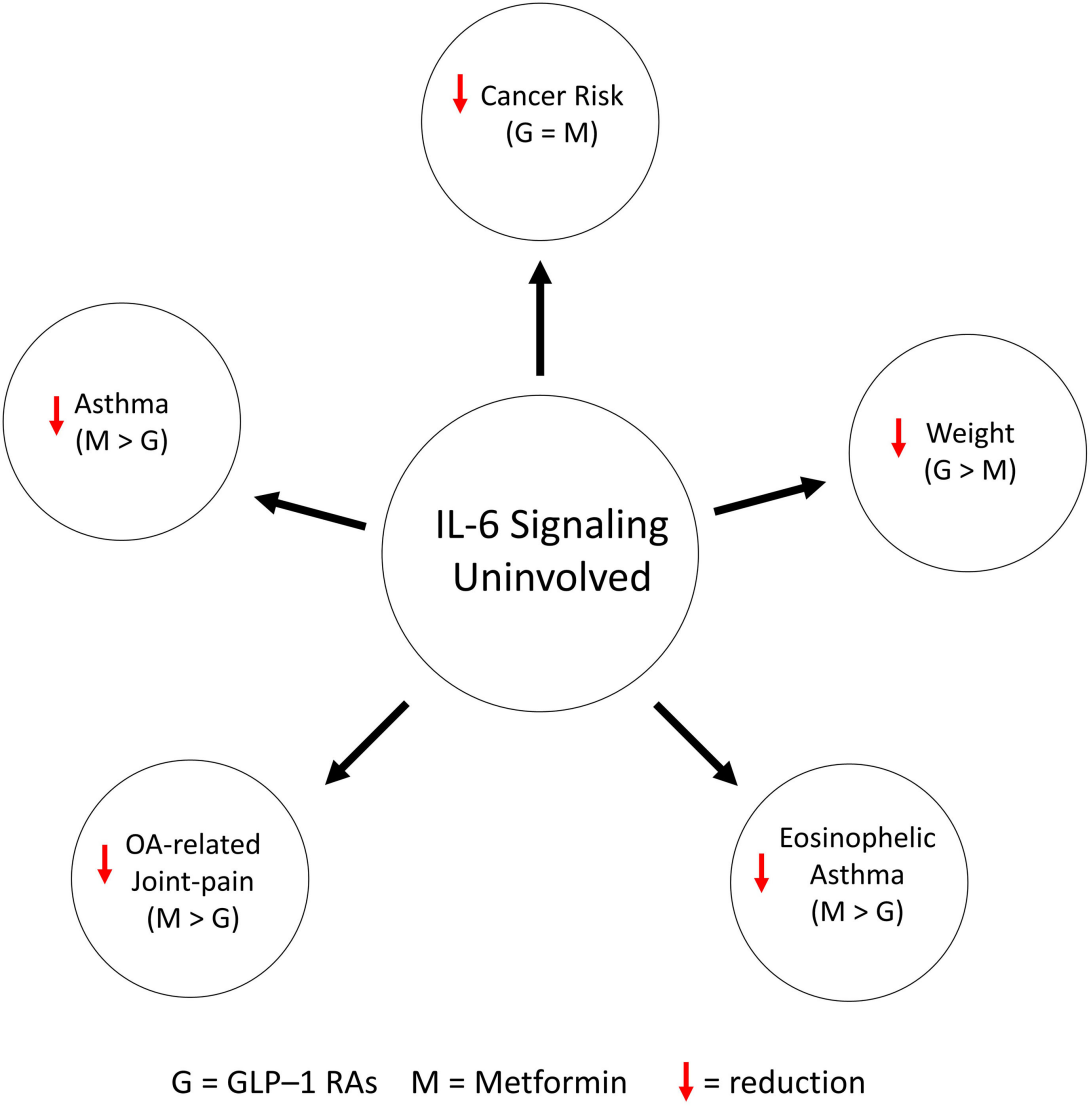
Effects of GLP-1 RAs and metformin independent of IL-6 signaling across different health conditions. IL-6 signaling is not involved in osteoarthritis-related joint pain. Interference with the IL-6 pathway does not affect cancer risk. Approximately half of the patients with asthma exhibit the eosinophilic (Type 2-high) phenotype, a subtype in which IL-6 has no pathogenic role. Weight loss induced by GLP-1 RAs is largely mediated by central nervous system mechanisms, independent of IL-6 signaling. In all these conditions, the anti-inflammatory effects of GLP-1 RAs and metformin are comparable; however, their clinical efficacy varies by condition. For example, because metformin lacks central effects on appetite, satiety, and energy intake, it is less effective than GLP-1 RAs for weight reduction. In contrast, metformin is superior in relieving osteoarthritis-related joint pain whereas cancer risk is similar with both treatments. Although Metformin may be more effective than GLP-1 RAs in the general asthma population, but is not expected to be more effective in neutrophilic asthma,.

Overall, these findings suggest that the superiority of GLP-1 RAs over metformin in the treatment of hyperglycemia, insulin resistance (in PCOS), and in reducing the risks of cardiovascular events and periprosthetic joint infection is related to their more pronounced suppression of circulating IL-6 levels. In contrast, their superior effect on weight reduction—which is independent of the IL-6 signaling pathway—likely underlies their greater efficacy in improving MASH, PCOS, and in prolonging survival in cancer patients. IL-6 pathway inhibition did not affect cancer risk in patients with rheumatoid arthritis treated with tocilizumab. Consistently, the risk of malignancy in T2D patients treated with GLP-1 RAs was similar to that observed in T2D patients treated with metformin. IL-6 signaling is also not implicated in osteoarthritis-related joint pain or in eosinophilic asthma. Accordingly, GLP-1 RAs were not superior to metformin in alleviating osteoarthritis-related pain or asthma in the general asthma population, in which over 70% of patients exhibit the eosinophilic phenotype (**Figure 3**).

Suppression of circulating IL-6 levels is proposed as one factor contributing to the superiority of GLP-1 RAs over metformin in the management of hyperglycemia, insulin resistance (in PCOS), neutrophilic asthma, and in reducing the risks of cardiovascular events and periprosthetic joint infection. Conversely, IL-6 suppression has also been proposed as a potential contributor to the allergic adverse events associated with GLP-1 RAs but not with metformin. Therefore, it is important to assess the strength of the evidence supporting the pathogenic role of IL-6 in these conditions and the underlying mechanisms involved. **Table 6** summarizes the available evidence.

**Table 6:** Strength of evidence supporting the pathogenic role of IL-6 signaling in various health conditions and the adverse effects of its impairment on allergic responses.

Condition	IL-6 mediated mechanisms (major)	Evidence strength	Supportive evidence	Conflicting evidence
Hyperglycemia	IL-6 activates JAK/STAT signaling and downstream inflammatory kinases (246). It also stimulates acute-phase protein production (247) and SOCS3 expression (235), potentially contributing to impaired glucose homeostasis.	Moderate	Two retrospective single-arm pre–post intervention studies of IL-6 blockade in RA patients (one large and one small) reported improvements in glycemic parameters (54, 55)	IL-6 has been reported to increase the expression and activity of insulin-degrading enzyme, suggesting that some metabolic effects may be mediated through altered insulin clearance rather than solely through induction of insulin resistance (248).
Insulin resistance	IL-6 activates JAK/STAT signaling and downstream inflammatory pathways (246), and induces SOCS3 expression, which interferes with insulin signaling (235).	Moderate	Two small single-arm pre–post interventional studies of IL-6 blockade in RA patients demonstrated improved insulin sensitivity (52, 53).	Although chronic IL-6 exposure is generally associated with hepatic insulin resistance, acute IL-6 signaling has been reported to enhance glucose uptake and improve insulin sensitivity in skeletal muscle under certain physiological conditions, such as exercise (29).
Cardiovascular events	IL-6 promotes endothelial dysfunction, monocyte/macrophage recruitment, foam-cell formation, plaque progression, and plaque destabilization (106). It also induces acute-phase reactants such as CRP (247).	High	Anti-IL-6 therapy reduced vascular inflammation and atherosclerosis-related endpoints in two double-blind, randomized, placebo-controlled phase II trials (107, 108).	Acute, short-term IL-6 signaling may exert cardioprotective effects (249). In addition, IL-6 has demonstrated atheroprotective effects in some experimental models (106), suggesting context-dependent functions.
Periprosthetic joint infection	IL-6 may contribute indirectly through promotion of hyperglycemia and insulin resistance, both of which are established risk factors for postoperative infection (211).	Moderate	The association between metabolic syndrome and post-operative infections is well established (250)	Despite the proposed detrimental metabolic effects of IL-6, infections were among the most common adverse events and serious adverse events reported during long-term IL-6 inhibitor therapy in RA patients (251).
Neutrophilic asthma	IL-6 promotes Th17 differentiation (85). Th17-derived IL-17 contributes to neutrophil recruitment and neutrophilic airway inflammation (87).	Low-Moderate	*In vitro* studies in human T cells demonstrate a role for IL-6 in Th17 differentiation (85), while the role of IL-17 in neutrophilic airway inflammation is well established (87).	Some studies have failed to demonstrate an association between obesity and neutrophilic airway inflammation in adults with asthma (252), challenging the proposed IL-6–obesity–Th17–neutrophilic asthma axis.
Allergy	Defective IL-6 signaling promotes Th2 skewing and allergic responses. IL-6 suppresses Th2 polarization through SOCS3-dependent inhibition of IL-2 signaling (235). Key manifestations of hyper-IgE syndrome have been linked to impaired IL-6 signaling (253).	High	A substantial body of clinical evidence indicates that loss-of-function mutations affecting IL-6 signaling, including mutations in IL6R, GP130, and STAT3, are associated with increased Th2 bias (235). Allergic adverse events are also commonly reported during IL-6 inhibitor therapy (**Table 6**).	Not available

CRP, C-reactive protein; GP130, glycoprotein 130; IL-6, interleukin-6; IL6R, interleukin-6 receptor; JAK, Janus kinase; RA, rheumatoid arthritis; STAT3, signal transducer and activator of transcription 3; SOCS3, suppressor of cytokine signaling 3; Th2, T helper type 2; Th17, T helper type 17.

By **Table 6**, the evidence supporting a pathogenic role for IL-6 is of low-to-moderate certainty in neutrophilic asthma, moderate certainty in hyperglycemia, insulin resistance, and periprosthetic joint infection, and high certainty in cardiovascular events and allergy.

It is also important to assess the strength of the evidence supporting the relative efficacy of GLP-1 RAs and metformin in improving various clinical conditions. The results are presented in **Table 7**.

**Table 7:** strength of the evidence supporting the relative efficacy of GLP-1 RAs and metformin in improving various clinical conditions.

Disease/condition	Comparative efficacy	Study description	Evidence strength	Strength/limitations
HbA1c (in T2D)	GLP-1 RAs ≥ Metformin (193)	Nationwide Danish register-based matched cohort study comparing GLP-1 RA and metformin initiators with HbA1c ≥42 mmol/mol.	Moderate	Strengths: Large sample size, nationwide registry data, matched comparison groups. Limitations: Observational design with potential residual confounding.
Insulin resistance (in PCOS)	GLP-1 RAs > Metformin (194, 196)	Two meta-analyses of RCTs in overweight/obese women with PCOS (194, 196)	Low - moderate	Strengths: Meta-analyses of randomized studies. Limitations: Small studies, low-to-moderate quality evidence; potential bias in some included studies.
Obesity	GLP-1 RAs > Metformin (4, 167, 168)	Phase 3 Wegovy trial, review of metformin studies, and active-controlled RCT comparing exenatide and metformin.	Moderate	Strengths: Large phase 3 trial, randomized active-comparator trial, consistent findings across studies. Limitations: Comparison partly relies on separate studies rather than direct head-to-head evidence.
MASH	GLP-1 RAs > Metformin (168, 195)	Two randomized active-comparator trials comparing exenatide or liraglutide with metformin in patients with T2D and NAFLD/MASH.	Moderate	Strengths: Direct randomized comparisons. Limitations: Modest sample sizes and short follow-up.
Polycystic ovary syndrome	GLP-1 RAs > Metformin (194, 196)	Two meta-analyses of RCTs, demonstrating superiority of GLP-1 RAs in reducing insulin resistance and body weight.	Low-moderate	Strengths: Evidence synthesized from randomized studies. Limitations: Small trials and low-to-moderate quality evidence.
Asthma	Metformin > GLP-1 RAs (197)	Retrospective active-comparator new-user cohort study using a Japanese national administrative database.	Moderate	Strengths: Large sample size (n=137,173), active comparator, new-user design. Limitations: No randomization, limited clinical detail, moderate risk of bias.
Osteoarthritis (joint pain)	Metformin > GLP-1 RAs (198)	Systematic review and network meta-analysis of RCTs evaluating antidiabetic drugs for knee osteoarthritis.	Moderate - high	Strengths: RCTs only, systematic search, generally high methodological quality. Limitations: Heterogeneity among studies and relatively small evidence base.
Osteoarthritis (perioperative)	GLP-1 RAs > Metformin (199, 200)	Two retrospective propensity score-matched cohort studies evaluating outcomes after THA/TKA.	Moderate	Strengths: Large databases, active comparator, propensity score matching. Limitations: Retrospective design, possible coding errors, lack of detailed glycemic control data (HbA1c/fructosamine).
Cardiovascular pathology	GLP-1 RAs > Metformin (157, 201)	Comparison of two large systematic reviews/meta-analyses of RCTs evaluating GLP-1 RAs versus placebo/control and metformin versus control.	Low	Strengths: Very large sample sizes (n1 = 99,599; n2 = 19,990), rigorous methodology, GRADE and advanced statistical analyses. Limitations: Indirect comparison, different populations and datasets, no head-to-head comparison.
Cancer risk	GLP-1 RAs ≈ Metformin for most cancers; GLP-1 RAs associated with higher kidney cancer risk (202)	Nationwide retrospective EHR-based cohort study including 1.65 million patients with T2D.	Moderate	Strengths: Very large, nationally representative cohort with standardized analyses. Limitations: Observational design, residual confounding, exposure misclassification, inability to infer causality.
Cancer progression	GLP-1 RAs > Metformin in cancer patients with T2D (203)	Retrospective propensity-matched cohort study using the TriNetX EHR database comparing GLP-1 RA and metformin users with T2D and cancer.	Moderate	Strengths: Large multicenter database, active comparator, propensity matching. Limitations: Retrospective design, incomplete cancer staging and treatment details, possible misclassification, residual confounding.

EHR, electronic health record; GLP-1 RAs, glucagon-like peptide-1 receptor agonists; HbA1c, glycated hemoglobin A1c; IR, insulin resistance; MASH, Metabolic dysfunction-associated steatohepatitis; NAFLD, non-alcoholic fatty liver disease; PCOS, polycystic ovary syndrome; RCT, randomized controlled trial; T2D, Type 2 diabetes; THA, total hip arthroplasty; TKA, total knee arthroplasty;.

By **Table 4**, the evidence supporting the conclusion of GLP-1 RA superiority over metformin in reducing cardiovascular pathology is of low certainty. The evidence supporting its superiority in PCOS and in insulin resistance (in PCOS patients) is of low-to-moderate certainty. The results for the other nine conditions are supported by higher certainty of evidence.

**Figure 4:**
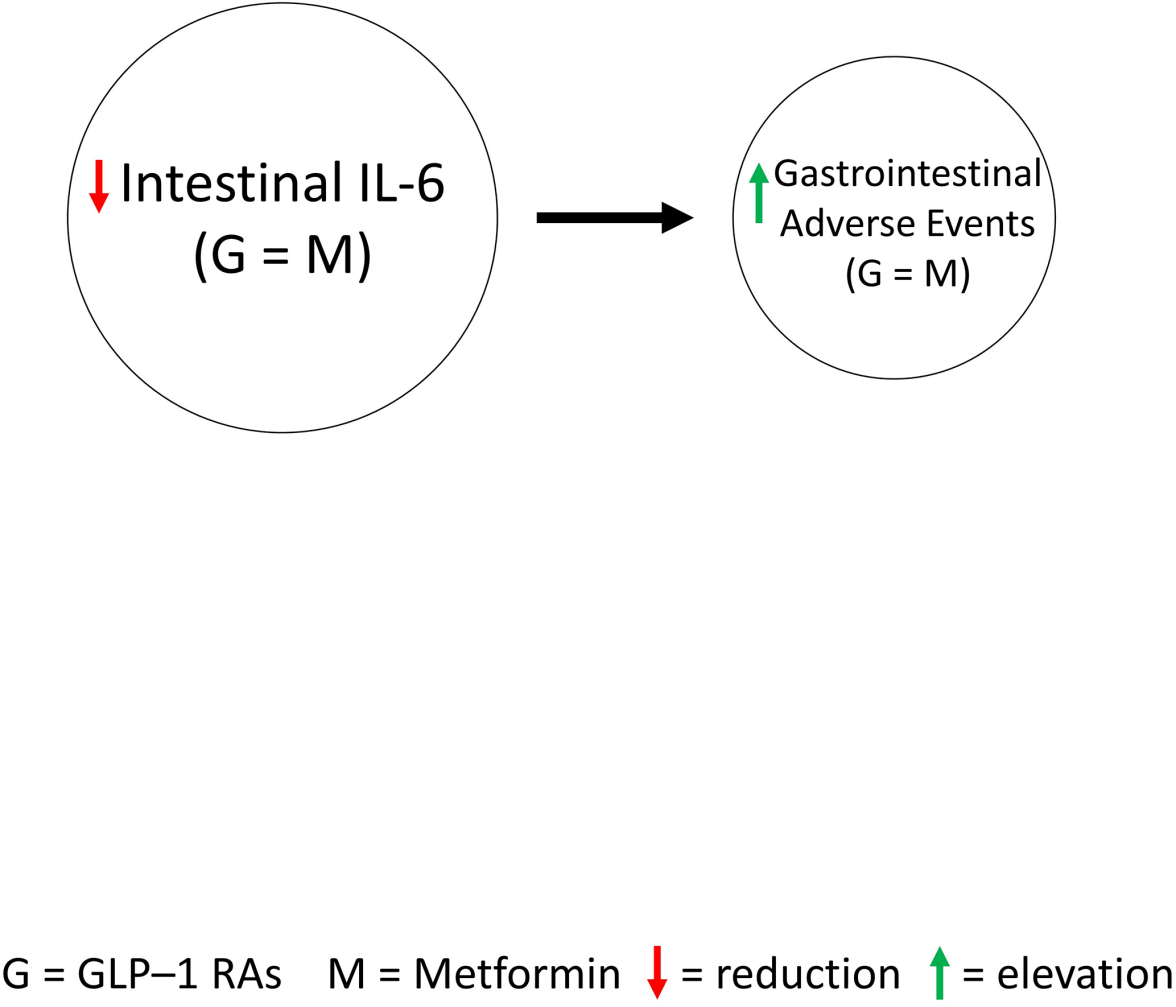
Effects of IL-6 suppression in the gastrointestinal tract on gastrointestinal adverse events. Both GLP-1 RAs and metformin, similar to IL-6 pathway inhibitors, interfere with local gastrointestinal IL-6 signaling and are therefore commonly associated with gastrointestinal adverse events. Interference of metformin with gastrointestinal IL-6 signaling has been demonstrated in mice with induced colitis, in fed old mice, and in human esophageal squamous cell carcinoma cell line. A group of colon adenocarcinoma patients identified from TCGA who demonstrated metformin gene signature, also showed decreased IL-6 pathway signaling. In addition, metformin has shown to downregulate IL-6 signaling in other human epithelial tissues such as ovarian cancer cells.

Suppression of IL-6 activity is proposed to underlie the frequently observed gastrointestinal adverse events associated with GLP-1 RAs, metformin, and several IL-6 pathway inhibitors (**Figure 4**). In contrast, the very rare occurrence of hypersensitivity reactions with metformin—unlike GLP-1 RAs and IL-6 pathway inhibitors —is likely attributable to its lack of effect on *circulating* IL-6 levels in patients with T2D. The question of why sarilumab, an IL-6 receptor inhibitor, rarely presents gastrointestinal or hypersensitivity adverse effects (**Table 6**) remains open. Sarilumab binds IL-6Rα *in vitro* with 15- to 22-fold higher affinity than tocilizumab, another IL-6 receptor inhibitor (254). However, whether this stronger receptor binding contributes to its distinctive adverse-effect profile is unclear.

Collectively, the integration of diverse data sources underscores the importance of IL-6 signaling in the distinct effects of GLP-1 receptor agonists and metformin across diverse disease states.

## Summary

Focusing on the roles of IL-1β, IL-6, and TGF-β1, this article compares the efficacy of GLP-1 receptor agonists (GLP-1 RAs) and metformin in the management and prevention of diverse diseases, their associated adverse effects, and their influence on cancer risk and survival. Stronger suppression of circulating IL-6 likely contributes to the superior efficacy of GLP-1 RAs in the treatment of hyperglycemia, insulin resistance (in PCOS), and in reducing the risks of cardiovascular events, and periprosthetic joint infection, but not to their weight-reducing effects. The superior effect of GLP-1 RAs on weight reduction, which is unrelated to the IL-6 signaling pathway, likely underlies their higher efficacy in improving MASH, PCOS and in prolonging survival of cancer patients. IL-6 pathway inhibition does not affect cancer risk. Consistent with this, the risk of malignancy in patients with T2D treated with GLP-1 receptor agonists was similar to that observed in those treated with metformin. IL-6 signaling is likewise not implicated in osteoarthritis-associated joint pain or in eosinophilic asthma. Accordingly, GLP-1 receptor agonists were not superior to metformin in alleviating osteoarthritis-related pain. Similarly, GLP-1 RAs were not superior to metformin in improving outcomes in the general asthma population, in which the eosinophilic phenotype predominates. Enhanced systemic IL-6 suppression may also help explain the higher frequency of allergic adverse reactions observed with GLP-1 RAs compared with metformin, whereas shared interference with IL-6 signaling in the gastrointestinal tract likely accounts for the common gastrointestinal side effects of both agents. Although metformin appears more effective in improving asthma outcomes in the general asthmatic population, GLP-1 RAs may offer advantages in the neutrophilic asthma subtype. Finally, the greater glucose-lowering and weight-reducing effects of GLP-1 RAs may underlie their superior survival benefits in patients with concurrent T2D and cancer, likely independent of IL-6 suppression.

## Data Availability

The original contributions presented in the study are included in the article/supplementary material. Further inquiries can be directed to the corresponding author.
